# Linkage Disequilibrium-Based Quality Control for Large-Scale Genetic Studies

**DOI:** 10.1371/journal.pgen.1000147

**Published:** 2008-08-01

**Authors:** Paul Scheet, Matthew Stephens

**Affiliations:** 1 Center for Statistical Genetics, Department of Biostatistics, University of Michigan, Ann Arbor, Michigan, United States of America; 2 Departments of Human Genetics and Statistics, University of Chicago, Chicago, Illinois, United States of America; University of Alabama at Birmingham, United States of America

## Abstract

Quality control (QC) is a critical step in large-scale studies of genetic variation. While, on average, high-throughput single nucleotide polymorphism (SNP) genotyping assays are now very accurate, the errors that remain tend to cluster into a small percentage of “problem” SNPs, which exhibit unusually high error rates. Because most large-scale studies of genetic variation are searching for phenomena that are rare (e.g., SNPs associated with a phenotype), even this small percentage of problem SNPs can cause important practical problems. Here we describe and illustrate how patterns of linkage disequilibrium (LD) can be used to improve QC in large-scale, population-based studies. This approach has the advantage over existing filters (e.g., HWE or call rate) that it can actually reduce genotyping error rates by automatically correcting some genotyping errors. Applying this LD-based QC procedure to data from The International HapMap Project, we identify over 1,500 SNPs that likely have high error rates in the CHB and JPT samples and estimate corrected genotypes. Our method is implemented in the software package fastPHASE, available from the Stephens Lab website (http://stephenslab.uchicago.edu/software.html).

## Author Summary

In large-scale studies of population genetic data, particularly genome-wide association studies, considerable effort may be spent on quality control (QC) to ensure genotype data are accurate. Typically, QC steps are applied independently to individual marker loci, with data from suspicious loci being excluded from subsequent analyses. Here we present a new QC tool, which exploits the fact that correlation of alleles among nearby genetic loci (linkage disequilibrium; LD) provides a certain amount of redundancy in genotype information, and that high rates of genotyping error at a marker may leave their trace in unusual patterns of LD. The method (a) aids in the detection of SNP loci with possibly elevated levels of genotyping error, and (b) in some cases allows for the correction of erroneous genotype calls, thereby salvaging some of the genotype data from the QC filtering process. We confirm on data from real populations that SNPs identified by this approach do show evidence for containing actual genotyping errors, and we also examine genotype intensity plots to confirm that many individual genotypes corrected by the method do appear to be called in error. More generally, these results demonstrate the potential utility of incorporating LD information into algorithms for processing and analyzing population genotype data.

## Introduction

Data quality has been implicated as a source of bias and loss of power in both linkage analyses and population-based association studies [1],[2],[3],[4]. Quality control (QC) is thus a critical step in large-scale studies of genetic variation. While, on average, high-throughput single nucleotide polymorphism (SNP) genotyping assays are now very accurate, the errors that remain tend to cluster into a small percentage of “problem” SNPs that exhibit unusually high error rates. Because most large-scale studies of genetic variation are searching for phenomena that are rare (e.g. SNPs associated with a phenotype), even this small percentage of problem SNPs can cause important practical problems. To alleviate these problems attempts are made to identify, and usually remove, problem SNPs before proceeding to a full analysis. However, while for pedigree studies considerable attention has been given to development of methods for detecting genotyping errors [5],[6],[1],[7], in population genetic studies rather simple QC filters are typically employed (e.g. removing SNPs with a high proportion of missing data, or showing very extreme deviations from Hardy–Weinberg equilibrium [8]; HWE).

Here we describe and illustrate how patterns of linkage disequilibrium (LD) can be used to improve QC in large-scale population-based studies. Intuitively, the method exploits the fact that LD among nearby markers provides built-in redundancy, allowing genotypes at a SNP to be called not only from the experimental data at that SNP, but also using data at nearby, correlated, SNPs. The result is a QC procedure that can not only identify individual SNPs that potentially have high genotyping error rates, but also automatically correct some incorrect genotypes.

## Results

We developed an LD-based QC procedure by modifying an existing statistical model for LD among multiple tightly-linked SNP markers [9] to allow for genotyping error. In brief, this existing statistical model captures patterns of LD in a population by assuming that each sampled haplotype resembles a mosaic of a (typically small) number of “base” haplotypes. The use of a relatively small number of base haplotypes allows the model to capture the limited haplotype diversity over small regions that is typical of many natural populations, while the mosaic assumption allows the model to capture breakdown in LD with genetic distance. The original version of this model assumed observed genotypes to be error-free. Here, to allow for, detect, and correct genotyping errors we modify this model by introducing a “genotyping error rate” parameter at each SNP, and develop statistical methods to estimate these SNP-specific error rates from unphased genotype data (see Methods). In addition to providing an estimated error rate for each SNP, the approach provides for each genotype a probability that it is incorrect, and a probability distribution for the actual correct genotype.

We assessed the utility of LD-based estimates of genotyping error in two ways. First, we applied the method to (unfiltered) genotype data on parent-offspring trios from the International HapMap Project [10] (see Methods), and compared the LD-based error rate estimates with the number of Mendelian Inconsistencies (MIs) at each SNP. Second, we applied the method to genotypes obtained by using the Affymetrix Mapping 500K chip to genotype the HapMap samples, and compared the LD-based error rates with the number of discrepancies between the Affymetrix genotype calls and the calls in the non-redundant filtered HapMap database (see Methods). In these two comparisons, the number of MIs, and the number of discrepancies, provide some independent indication of the genotyping error rate at each SNP, against which our LD-based error rate estimates can be compared.

Overall the LD-based genotyping error rate estimates were similar in magnitude to estimates based on MIs and discrepancies. For the unfiltered HapMap data, the LD-based error rate estimate was 0.28% for CEU and 0.36% for YRI, slightly higher than the total rate of MI-causing genotyping errors (0.17% for CEU and 0.23% for YRI, assuming each trio containing an MI contains a single genotyping error), possibly reflecting the fact that not all genotyping errors will cause an MI [11]. For the Affymetrix data, the LD-based error rate estimates were 0.24% for CEU, 0.22% for JPT+CHB, and 0.44% for YRI, similar to the average discrepancy rates (0.29% in CEU and JPT+CHB; 0.38% in YRI). (Note that, since up to half of the discrepancies are likely to be due to errors in the HapMap, rather than Affymetrix, data, the LD-based error rate estimates suggest slightly higher error rates than do the discrepancy data.)

**Figure 1:**
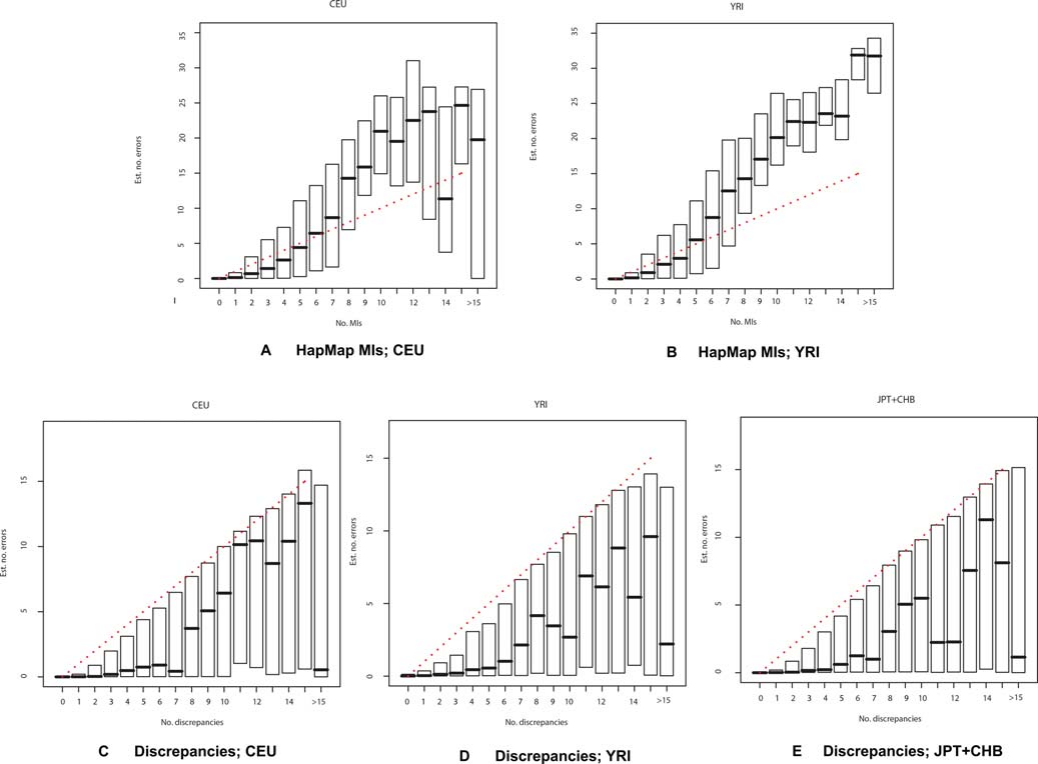
SNP-specific estimates of number of errors based on LD correlate with number of MIs and discrepancies. Each plot contains a box corresponding to the number of observed MIs or discrepancies (horizontal axis). The position of the bottom and top of a box relates the first and third quartiles of the estimated number of MIs or discrepancies (vertical axis), with the median displayed as a horizontal line in the middle of each box. The red dotted line indicates equality between the number of estimated errors and observed MIs or discrepancies. *First row (A-B)*: The total number of expected errors at each SNP, based on LD, was calculated for the HapMap data and plotted against the number of MIs. *Second row (C-E)*: The total number of expected errors at each SNP, based on LD, was calculated for the Affymetrix data, and plotted against the number of discrepancies between the Affymetrix and HapMap genotype calls. In general, the median and the upper quartile for the number of estimated errors increase with the number of discrepancies/MIs. The fact that the lower quartile is at 0 in (C-E), even for SNPs with many discrepancies, could partially reflect the existence of SNPs with many discrepancies, but with few errors in the Affymetrix data (the discrepancies being due to errors in the HapMap data).

More importantly, SNP-specific LD-based error rate estimates were positively correlated with number of MIs or discrepancies (Figure 1). In particular, SNPs with a large number of MIs/discrepancies also tended to have high LD-based error rate estimates. For example, in the Affymetrix data, among SNPs with at least a 10% discrepancy rate, 60% had an elevated LD-based error rate (>1%), whereas among SNPs with 0 discrepancies, only 5.7% had a similarly elevated LD-based error rate. Similarly, in the HapMap data, among SNPs with at least 9 MIs, 91% had an LD error rate >1%, whereas among SNPs with 0 MIs only 2% had LD error rate estimates exceeding this level.

These results demonstrate the potential for patterns of LD to help identify “problem” SNPs with very high error rates. We attempted to more fully quantify this potential, but these attempts were hindered by the fact that neither MIs nor discrepancies provide a completely satisfactory “gold standard” against which to compare. For example, MIs are not effective at identifying all genotyping errors, since many errors (e.g. miscalling homozygous parents as heterozygotes) do not lead to MIs. And while a discrepancy between two genotype calls implies an error in at least one of the calls, it does not indicate which of the calls is incorrect. We therefore undertook a more qualitative assessment, by visually examining higher-level data from the Affymetrix genotyping assay–specifically, plots of normalized intensities for each allele–for SNPs where our LD-based estimates disagreed most strongly with the numbers of discrepancies. (These intensity data are not generally available for the HapMap data.)

**Figure 2:**
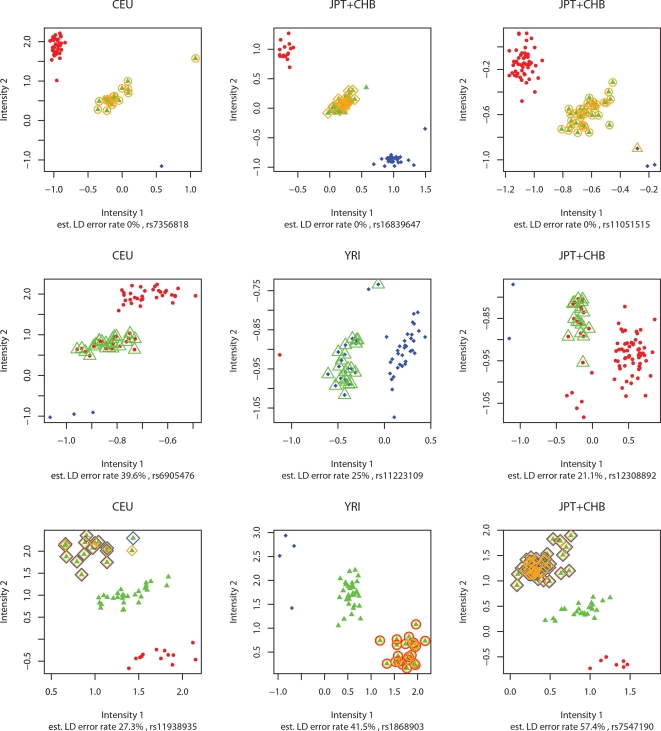
Example genotype intensity scatter plots from Affymetrix 500K technology on unrelated HapMap samples. Original calls from the Affymetrix data are indicated by colour and shape of the small solid points (homozygotes: blue♦, red •, heterozygotes: green▴). The larger, open symbols with the same colour scheme (◊, ○, Δ) represent corrected genotype calls from applying our LD-based method to the Affymetrix data. Orange symbols indicate genotypes that are discrepant between the Affymetrix and HapMap datasets, with the shape of these symbols indicating the genotype calls in the HapMap database. LD-based error rate estimates are those obtained from applying the LD-based method to the Affymetrix data. The first row shows plots for three SNPs with large numbers of discrepancies between HapMap and Affymetrix calls, but low LD-based error rate estimates and clean intensity plots, with three well-separated clusters. The likely explanation for these results is that the discrepancies are due to errors in the HapMap database, and not the Affymetrix calls on which the LD-based error rates are based. The second row shows plots for three SNPs where the HapMap and Affymetrix calls agree (0 discrepancies) but high LD-based error rate estimates and unusual intensity plots. The unusual intensity results, combined with the fact that genotypes identified as likely to be incorrect by the LD-based method tend to cluster together, suggests that the high LD-based error rates reflect genuine signal at these SNPs, such as genotyping errors or other anomalies (e.g. copy number variation). This illustrates the potential for the LD-based method to detect problems that duplicate genotyping may miss. The third row shows plots for three SNPs with high LD-based error rate estimates, and large numbers of discrepancies, where the intensity plots are relatively clean, but where the genotyping algorithm appears to have done a poor job of clustering the genotypes. In each case the LD-based method successfully identifies and corrects most of these erroneous genotypes. Although these examples were chosen to illustrate particular points, they are not atypical in that we saw other examples of each type of behaviour.

Among SNPs with large numbers of discrepancies, but low LD error rates, many of the Affymetrix intensity plots show three well-separated clusters with genotypes apparently correctly-called (Figure 2a). For example, for 50 JPT+CHB SNPs with 9 discrepancies but with LD error rates <1%, we judged, subjectively, that at least 23 showed relatively clean intensity plots, with little or no evidence of typing error. A natural explanation for this is that the discrepancies are due to errors in the HapMap database, rather than in the Affymetrix calls from which the LD-based error rates are computed. In contrast, among SNPs with 0 discrepancies but high LD-based error rates, many of the intensity plots failed to show well-separated clusters in the usual places, and several were suggestive of copy number variation (Figure 2b). Thus, our LD-based method appears, in some of these cases, to be picking up on meaningful problems with the genotype calls, despite the concordance between the Affymetrix calls and those from HapMap, obtained independently from different genotyping centers. For other SNPs, whose plots did exhibit three well-separated clusters in the expected places, it may be that the high LD-based error rate estimates are simply inaccurate. However, it is also possible that some of these SNPs are mis-mapped, since this could produce a high estimated LD-error rate. During PHASE II of the HapMap, 21,177 SNPs from PHASE I were identified as having an ambiguous position, or other signatures that suggest unreliability [12], and although these SNPs were not included in our comparison it is possible that some similar inaccuracies remain. We list approximately 600 SNPs with high LD error rate estimates but 0 discrepancies in Text S1.

**Figure 3:**
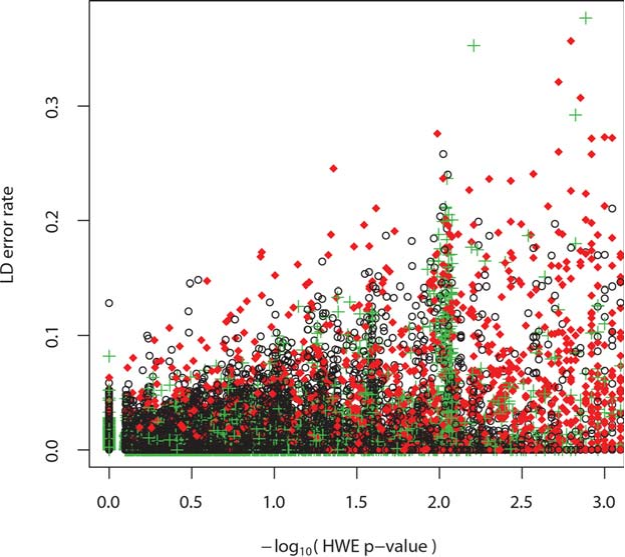
LD-based error rate vs. HWE. An LD-based error rate was estimated and a test of HWE (PEDSTATS [13]) was conducted for data from chromosome 7 HapMap CEU *unrelated* samples at each of the following types of SNPs: passed all HapMap QC criteria and had *zero* MIs (black ○); passed QC criteria with *exactly one* MI (green +); and failed due to the presence of *multiple* MIs (red ♦). SNPs which failed QC due to extreme deviations from HWE are excluded.

The above results illustrate the difficulty of assessing the accuracy of our LD-based error rate estimates. Even though the LD-based estimates sometimes disagree greatly with the duplicate genotyping results, it is unclear in what proportion of cases the LD-based estimates are inaccurate. The results also highlight the fact that the LD-based estimates can complement, rather than duplicate, other approaches to QC such as multiple rounds of genotyping. To further examine the extent to which the LD-based approach complements existing QC procedures, we compared LD-based error rate estimates with the results of testing SNPs for deviations from HWE, which is probably the most common current approach to QC in population studies. We found LD-based error rates and HWE test statistics to be relatively uncorrelated (Figure 3), although the subset of SNPs with the highest LD-based error rates overlaps moderately with the subset showing the most significant deviations from HWE: among the top 1% of SNPs in each category in the filtered (respectively unfiltered) data, 19% (respectively 42%) were shared.

**Figure 4:**
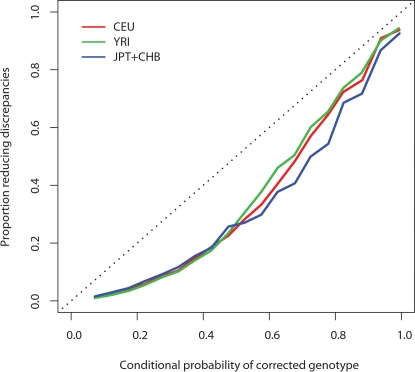
Calibration of conditional probabilities of corrected genotypes in the Affymetrix data. Separately in 3 HapMap populations and for all 22 autosomes, we calculated the conditional probabilities of genotypes other than the observed genotype calls. We then binned these probabilities and, within each bin, calculated the proportion of genotypes which, if switched to the most-probable genotype other than that observed, resulted in a decrease in the number of discrepancies with the HapMap calls.

The LD-based method has several advantages over HWE for performing QC: in addition to providing quantitative estimates of the error rate at each SNP, the LD-based method also estimates an error probability for each individual genotype, and can attempt to correct genotypes that it deems likely to be incorrect. To quantify its success at this we examined whether using our method to correct genotypes reduced the number of MIs/discrepancies, and indeed it did. Correcting HapMap CEU genotype calls reduced the number of MIs by 33% when parents and children were analysed together, ignoring the known relationships, and by 21% when parents and children were analysed separately. Correcting the Affymetrix 500K calls reduced discrepancies with HapMap by 13% for CEU samples, 8% for YRI and 11% for JPT+CHB. Furthermore, although the probabilities assigned to corrected genotypes were not completely well-calibrated, the reduction of discrepancies *was* appreciably greater for those corrections in which our method was most confident (Figure 4). One consequence of this is that one could further improve genotyping accuracy, at the expense of a slightly lower call rate, by treating genotype calls for which the assigned probability of error exceeds some threshold as “missing”. Alternatively, and perhaps preferably, one could take account of these probabilities in downstream analyses, using Bayesian statistical methods [14] to downweight the influence of genotypes in which one was less confident.

The fact that using LD to correct genotypes reduces both the number of MIs and the number of discrepancies suggests that it also reduces the overall genotyping error rate, and we attempted to quantify this reduction. However, this was again complicated by the fact that neither MIs nor discrepancies provide perfect gold standards against which to compare. In the case of discrepancies, a naive analysis, assuming that the error rates in the two data sets are equal (so half the discrepancies are due to errors in the Affymetrix data), and that each genotype error creates a discrepancy, would suggest that our method reduced genotyping error rates by 16-26%. However, we found several examples of SNPs where correcting genotypes with our method *increased* the number of discrepancies, but where visual examination of intensity plots suggested that the corrected genotype calls were likely correct, or at least more sensible than the original genotype calls. For example, consider the three SNPs with 0 discrepancies but high estimated LD error rate in Figure 2b. In all three cases our method makes many genotype corrections, and, strikingly, the genotypes it chooses to correct tend to cluster together in the intensity plots. Since our method does not take into account the intensity data in selecting which genotypes to correct this strongly suggests that the LD-based method is picking up on genuine anomalies in the underlying genotype calls, and not simply making mistakes in its corrections. However, despite this, in all three SNPs every corrected genotype *increases* the number of discrepancies in the data. Due to this type of effect the reduction in the number of discrepancies achieved by our method may underestimate the actual reduction in errors achieved, perhaps appreciably.

In the case of interpreting the reduction in MIs, there are different problems. In particular, there are many ways of reducing MIs that would actually increase the number of genotyping errors. For example, changing every parent at every SNP to be a heterozygote would completely remove all MIs, while presumably increasing the total number of genotype errors. However, if genotype changes of this type were being made randomly, independent of actual errors, then we would not expect to see an excess of genotype corrections being made in trio-SNP combinations with MIs. In fact, 37% of corrected genotypes occurred in a trio-SNP combination with an MI, whereas only 0.7% of trio-SNP combinations actually exhibit an MI. This provides strong indirect evidence that these corrections are actually correcting the genotyping error that lead to the MI, rather than simply randomly changing parents to be heterozygotes. Also, MIs in trio data can be caused by deletions, rather than simple genotyping error [15],[16]. Since our method does not explicitly model deletions it is perhaps unsurprising that it tended to correct genotypes less often in trios whose MIs were consistent with a deletion than in other trios: among trios with deletion-consistent MIs, 33% had at least one genotype corrected, compared with 50% among trios with other MIs.

For a practical application of our method, we applied it to the Chinese and Japanese analysis panels (CHB+JPT) in the filtered HapMap database. Because these panels do not include data on trios, the HapMap QC filter based on MIs could not be applied to these individuals, and so the filtered CHB+JPT data may be expected to contain more genotyping errors than the other panels. Applying the LD-based QC method to all 2.4 million polymorphic loci from the autosomal chromosomes of the 90 CHB+JPT individuals, we estimate an LD-based error rate of 0.13% and identify approximately 1,500 SNPs with an LD-based error rate greater than 15% (4,300 exceed 10%). Additionally, we provide over 200,000 individual genotypes that our method identifies as likely to be incorrect (specifically, for which the conditional probability of the observed genotype is less than that for a different genotype). We provide a complete list of SNPs and genotypes at lower error rates and probability thresholds in Text S1.

## Discussion

We have described and illustrated a novel method for using patterns of LD to improve QC in large-scale population studies. The method complements existing approaches to QC, and can find genotyping problems that other methods, including duplicate genotyping, may miss. Performance of the method will depend on several factors, including SNP allele frequency, and the amount of LD in the data, which typically increases with SNP density. The results we present here are based on relatively dense data (>500k markers genome-wide) on (mostly) common variants. However, we have also found the method capable of identifying SNPs with high error rates in substantially less dense data (e.g. the Illumina Human-1 112k bead chip). For whole-genome resequencing data we would expect performance to be even better for the common variants, due to the increased information, although the potential for LD to detect genotyping errors in very rare variants seems likely to be limited. While, inevitably, not all genotyping errors can be detected from patterns of LD, the use of LD information is essentially free, is practical for large data sets (in our implementation, application to 1,000 individuals typed at 500,000 SNPs would require about 270 hours on a single 3 GHz Intel Xeon processor), and has the advantage over tests for HWE that it is able to detect, and in many cases correct, individual genotyping errors. Our method has been implemented in the software package fastPHASE.

Patterns of LD have previously been recognized as an effective way to estimate missing genotypes [17],[9],[14],[18], and attempting to use LD to detect genotyping errors is, perhaps, a natural next step. However, there are many possible approaches to implementing this idea in practice (e.g. a recent paper [19] takes an approach rather different to the one we took here, based on applying the four-gamete test to pairs of SNPs in the data set). Our approach, which is based on introducing error-rate parameters into a statistical model for multi-locus genotype data, has several desirable features, including providing quantitative estimates of error rates, quantitative assessments of the probability that each individual genotype is wrong, and quantitative assessments of the probability of alternative genotypes to those that are called. Also, our method is “self-training”, in that it does not require a “gold-standard” set of data to establish normal patterns of LD, but rather establishes normal patterns of LD from the (imperfect and unphased) genotype data available. The model for LD that we used here is particularly well-suited to this purpose, because it can be fit efficiently to unphased genotype data, even when allowing for genotyping error. Not all models for LD enjoy this property. For example, the PAC model [20] provides a model for LD that is in some ways preferable to the one we used here, but is considerably harder to fit to unphased data (even without error), requiring more sophisticated and computationally-intensive algorithms. However, we note that in some cases it might be acceptable to treat a particular phased data set (e.g. the HapMap data) as an error-free gold standard, and use it to detect errors in other data sets [18]: in this case the PAC model would provide a viable alternative to our approach.

Since our primary motivation was to exploit LD to help detect markers with high genotyping error rates, our model allows error rates to vary across SNPs. In contrast, we have implicitly assumed equal error rates across individuals. In fact, due to issues such as DNA sample quality, some individuals may have higher error rates than others. We already estimate a large number of parameters in the model, and therefore have not attempted to relax this assumption here. However, this would be an interesting, and potentially useful, extension of this work.

In addition to detecting and correcting genotyping errors, our approach also lends itself to several other applications. In fastPHASE we have implemented two of these: testing for nonrandom missing data patterns, which may be of interest in genetic association studies where differential missingness patterns between groups can lead to spurious associations; and detecting “strand” errors, where the same SNP has been typed on two different platforms, which, perhaps unbeknownst to the investigator, are assaying different strands. This last application is particularly important for merging results from different studies performed on different platforms.

As described here, our approach works directly with discrete genotype calls, rather than with underlying intensity data used to obtain these calls. This has the advantage of making it independent of the genotyping platform used to obtain the data, and also making it applicable to data sets, such as the HapMap genotype database, where the intensities are not readily available. However, our approach could be readily modified to deal directly with the underlying intensity data, explicitly combining LD information with the intensity data to improve genotype calling accuracy [21]. From a purely statistical perspective one would expect such a one-stage procedure, when properly implemented, to outperform the two-stage procedure we adopt here. Further, intensity plots for the Affymetrix 500K data used in this study suggest that the benefits of incorporating both types of information could be considerable: it would allow patterns of LD to help identify cluster centers, and guide genotype calls, when the intensity data at a particular SNP are noisy, but downweight their influence at SNPs where intensity data are clean and unambiguous. Similarly, our approach could be combined with other types of higher-level data, such as assembled reads from whole-genome resequencing technologies. In these technologies, genotyping accuracy will be greatly influenced by the fold coverage available. We anticipate that effective use of LD information will reduce the coverage necessary to obtain a given level of genotyping accuracy, hence reducing the cost of future genome-wide studies of population genetic variation.

## Methods

### Data

The comparisons with MIs reported here were all performed by applying our method to unfiltered data from HapMap trios. Specifically, we used the CEU and YRI data from chromosome 7 (4 January, 2007; NCBI build 35), excluding SNPs that failed QC based on pass-rate (proportion of genotypes not marked as “missing”) and duplicate sample discrepancies. For the comparison with HWE we excluded SNPs which failed HapMap QC due to HWE (p-value <10^−4^), since, due to the popularity of HWE as a QC measure, SNPs showing extreme deviations from HWE are likely to be excluded from analyses. Unless otherwise stated, results are from applying our method separately to each sample of 90 individuals, ignoring the known parent-offspring relationships. This is because, although the method is designed for samples of unrelated individuals, we have found that it is also effective for data sets where individuals are related to one another, and applying it to all 90 individuals facilitates comparisons with MIs, since these are identified using data on all 90 individuals. In some cases we also report results obtained from applying the method separately to the parents and children.

The comparisons with discrepancies reported here were all obtained by applying our method to data on the unrelated HapMap individuals obtained using the Affymetrix 500k chip (http://www.affymetrix.com/support/technical/sample_data/500k_hapmap_genotype_data.affx). Specifically, we considered genotype data on the *unrelated* samples on all 22 autosomes, separately for each of the 3 HapMap analysis panels. To calculate the discrepancies, we compared the Affymetrix calls with data from the HapMap database (13 March, 2007; NCBI build 36). We excluded from this analysis those SNPs where HapMap calls were obtained from the same Affymetrix chip. To view the intensities of these SNPs, we obtained the intensities from the HapMap project website (http://www.hapmap.org/downloads/raw_data/affy500k/). Before plotting, we standardized each intensity value by subtracting the mean and dividing by the standard deviation of the intensities among all SNPs for the individual corresponding to that value (separately for each chip, NSP and STY). Note that although this simple standardization strategy appeared to suffice for our purposes, more sophisticated strategies are generally performed by the best genotype calling algorithms.

For a practical application of our method, we applied it to data on the combined CHB+JPT HapMap genotypes from the HapMap database (forward strand; 13 March, 2007; NCBI build 36). We provide a complete list of SNPs with estimated LD error rates, as well as individual genotypes where the conditional probability of the observed genotype was less than 0.95).

### Incorporating Genotyping Error into a Model for LD

We incorporated a genotyping error component into a previously-described model for multi-locus LD [9]. To briefly review this model, let 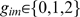 denote the observed unphased genotype for individual *i* (1,…, *n*) at marker *m* (1,…, *M*). The model in [9] assumes that the genotypes from each individual, along each chromosome, derive from a hidden Markov model (HMM). Specifically, at each SNP, each observed allele is assumed to derive from one of *K* haplotype *clusters* (states in the HMM), each of which has its own cluster-specific allele frequencies (emission probabilities), the set of which is denoted by *θ*. Thus, for unphased data, each observed genotype is assumed to derive from 2 (not necessarily distinct) clusters. To model the LD among nearby SNPs, cluster memberships are assumed to change gradually along each haplotype, specifically according to a Markov process whose jump probabilities are controlled by a parameter *r*; conditional on a jump at *m*, cluster *k* (1,…, *K*) is chosen with probability *α_km_*.

Since the clusters (HMM states) from which each allele is derived are unobserved, the probability of the genotypes for individual *i* is obtained by summing over all possible values for these latent variables:
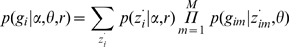
(1)where 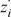 denotes the vector of latent cluster memberships for individual *i*. Conditional on the parameters of the model, genotypes from different individuals are assumed to be independent, and so the likelihood is obtained by multiplying together (1) across individuals. See [9] for further details, including methods for computing this likelihood efficiently, and for estimating the parameters of this model by maximum likelihood via the EM algorithm.

Here, we modify this model by letting 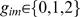 denote the *observed* unphased genotype for individual *i*, and introducing further latent variables *x_im_* to denote the corresponding *true* genotype. We assume that genotypes *g* are observed, possibly with error, according to some model *p*(*g* | *x*, *ε*), given below, where *ε* represents an error rate (or vector of rates). The term 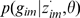 in (1) is replaced by a sum:
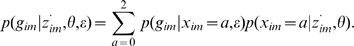
(2)


We apply an efficient algorithm for calculation of this likelihood based on Baum-Welch algorithms for HMMs (Text S1).

### Error Model

To obtain our results, we restricted attention to a particular error model, represented by the transition probability matrix in Table 1. We allow *ε* to vary by SNP marker, so that *ε* = (*ε*_1_,…, *ε_M_*), where *ε* = (1,…, *M*) is itself a vector of rates. Conditional on the model parameters, errors are assumed to occur independently across sites and across individuals. This particular model does not allow for the observation of a homozygote of one allelic type when the true genotype is a homozygote of the other type, since we expect this type of error to be relatively rare with current genotyping technologies. However, we did briefly explore various error models, including those which do allow this type of error (Text S1).

**Table 1:** Error model represented as a transition probability matrix.

		observed, *g*		
		0	1	2
	0	1−*ε* ^0^	*ε* ^0^	0
true, *x*	1	*ε* ^10^	1−(*ε* ^10^+*ε* ^12^)	*ε* ^12^
	2	0	*ε* ^2^	1−*ε* ^2^

Each cell contains the probability that a particular genotype *g* is observed, given the true genotype *x* is present. Superscripts denote elements of a vector of error rates *ε*, where 0<*ε* ^0^<*ε* ^2^<1 and 0<*ε* ^10^+*ε* ^12^<1.

### Parameter Estimation

For (*α*, *θ*, *r*), we attempt to obtain maximum likelihood (ML) estimates via an EM algorithm (Text S1). We fixed the number of clusters (*K*) to be 12 for the analysis of HapMap data. This choice was based on cross-validation results (for imputing missing genotypes) over a range of convenient possibilities of *K*. We also considered smaller values (Table 1 in Text S1). For *ε* we found that obtaining maximum likelihood estimates was not the best approach. Note that genotyping assays are, for most SNPs, very accurate, and so, a priori, values of *ε* are expected to be near 0. Because maximum likelihood estimation does not take this prior information into account, it tended to produce too many non-zero estimates of *ε*. To alleviate this problem we took the approach of putting a prior distribution on *ε*, with a mode at 0, and estimating *ε* using the maximum a posteriori (MAP) estimates. To facilitate computation we chose priors that were Beta (*a*,*b*) for the homozygote error rates *ε* ^0^ and *ε* ^2^, and Dirichlet (*a*,*b*,*a*) for the heterozygous error rates (*ε* ^0^, 1, –*ε* ^10^, –*ε* ^12^, *ε* ^12^). With these priors it is straightforward to obtain the MAP estimates using the EM algorithm. We compared results across three different values of (*a*,*b*) = (1,1), (0.9,2) and (0.9,2); the first of these corresponds to a uniform prior, and so the MAP estimates are the maximum likelihood estimates; the second and third produce increasingly strong shrinkage of estimated error rates towards 0. Although these comparisons are far from comprehensive, the results (Table 1) suggested that (*a*,*b*) = (0.9,2) provides a useful tradeoff between shrinking *ε* towards 0 and still identifying SNPs with high values of *ε*. In contrast, (*a*,*b*) = (0.9,2)seemed to shrink error rate estimates too much towards 0, resulting in very few genotypes being corrected; and, as noted above, the maximum likelihood estimates ((*a*,*b*) = (1,1)) tended to produce too many non-zero estimates of *ε*, and as a result corrected too many genotypes (actually increasing the number of discrepancies between HapMap and Affymetrix calls).

### Error Detection and Correction

We calculate an LD-based SNP-specific expected number of genotype errors by summing the conditional probabilities of incorrect genotype calls across all individuals at a particular SNP *m* as follows:
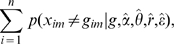
(3)where 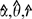 and 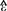 are estimates from the EM algorithm. Reported SNP-specific LD-based genotyping error rates are obtained by forming the ratio of this sum (3) to the number of observed (nonmissing) genotypes at SNP *m*. Reported overall LD-based genotyping error rates are obtained by summing both the numerator and denominator of this ratio across SNPs, and forming the ratio of these sums.

Conditional probabilities of individual genotypes are used to impute corrected genotype calls. Specifically, a genotype for individual *i* at marker *m* may be corrected if
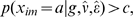
for an alternate genotype *a*≠*g_im_* and some probability threshold *c*. To obtain our results we set *c* equal to 0.5.

## Supporting Information

Text S1 Supporting information for Linkage disequilibrium-based quality control for large-scale genetic studies. Appendix for methods; comparisons of different priors and error models; list of SNPs and corrected genotypes from International HapMap Project database; large versions of figures.
(0.36 MB PDF)
