# Supplementary material for: Linkage Disequilibrium-Based Quality Control for Large-Scale Genetic Studies
Source: PLoS Genet. 2008 Aug 1;4(8):e1000147. doi: 10.1371/journal.pgen.1000147 (PMC2475504; doi:10.1371/journal.pgen.1000147)
Supplement: Text S1 — Supporting information for Linkage disequilibrium-based quality control for large-scale genetic studies. Appendix for methods; comparisons of different priors and error models; list of SNPs and corrected genotypes from International HapMap Project database; large versions of figures. (0.36 MB PDF) [file pgen.1000147.s001.pdf]

Text S1 for

Linkage disequilibrium-based quality control for large-scale genetic studies

Paul Scheet* and Matthew Stephens

July 1, 2008

Contents

1 Forward and Backward Algorithms 2

2 EM Algorithm 4

3 Sensitivity to choice of prior and number of clusters 6

4 Sensitivity of alternate error models 7

5 HapMap SNPs and Corrected Genotypes 9

6 Large Version of Figures 10

*email: PSCHEET@ALUM.WUSTL.EDU

1 Forward and Backward Algorithms

Here we give the details of an EM algorithm which jointly estimates $(\alpha, \theta, r, \epsilon)$ for a simple error model, given below. The EM algorithm involves iteratively calculating expected values for latent variables, given a current value of the parameters, and then updating the parameter estimates based on the expectations computed previously. We first derive the forward and backward algorithms for the model with genotyping error. These are necessary for subsequent calculations. For simplicity, we let ν denote (α, θ, r).

The forward and backward probabilities $\phi^i_{\nu,\epsilon}$ and $\beta^i_{\nu,\epsilon}$ are given by

$$\begin{aligned} \phi^i_{\nu,\epsilon}\big(m, \{k_1, k_2\}\big) &:= p\big(g_{i1}, \ldots, g_{im}, z^{\cdot}_{im} = \{k_1, k_2\}|\nu, \epsilon\big)\left(\frac{1}{2}\right)^{I_{\{k_1 \neq k_2\}}} \text{ and} \\ \beta^i_{\nu,\epsilon}\big(m, \{k_1, k_2\}\big) &:= p\big(g_{i(m+1)}, \ldots, g_{iM}|z^{\cdot}_{im} = \{k_1, k_2\}, \nu, \epsilon\big), \end{aligned}$$

where $\nu = (\alpha, \theta, r)$ and $I_{\{A\}}$ is the indicator function, equal to one if A is true and zero otherwise. Let j_{im} denote the number jumps (to new clusters) in the interval between markers $m-1$ and m for individual i, with

$$p\big(j_{im} = a|r\big) = \begin{cases} (1-r_m)^2, & a = 0, \\ 2r\,(1-r_m), & a = 1, \\ r_m^2, & a = 2. \end{cases}$$

The forwards calculation is given by

$$\begin{aligned} \phi^i_{\nu,\epsilon}\left(m+1, \{k_1, k_2\}\right) = p\big(g_{i(m+1)}|z^{\cdot}_{i(m+1)} = \{k_1, k_2\}, \nu, \epsilon\big)\Big[p\big(j_{im} = 0|\nu\big)\phi^i_{\nu,\epsilon}\big(m, \{k_1, k_2\}\big) \\ + \frac{p\big(j_{im} = 1|\nu\big)}{2}\Big(\alpha_{k_1(m+1)} \sum_{k'=1}^{K} \phi^i_{\nu,\epsilon}\big(m, \{k', k_2\}\big) \\ + \alpha_{k_2(m+1)} \sum_{k'=1}^{K} \phi^i_{\nu,\epsilon}\big(m, \{k', k_1\}\big)\Big) \\ + p\big(j_{im} = 2|\nu\big)\alpha_{k_1(m+1)}\alpha_{k_2(m+1)} \sum_{k'_1=1}^{K}\sum_{k'_2=1}^{K} \phi^i_{\nu,\epsilon}\big(m, \{k'_1, k'_2\}\big)\Big], \end{aligned}$$

for $m = 1, \ldots, M-1$, where $\phi^i_{\nu,\epsilon}\big(1, \{k_1, k_2\}\big) := p\big(g_{i1}|z^{\cdot}_{i1} = \{k_1, k_2\}, \nu, \epsilon\big)\alpha_{k_1 1}\alpha_{k_2 1}$ and

$$p\big(g_{im}|z^{\cdot}_{im} = \{k_1, k_2\}, \theta, \epsilon\big) = \sum_{a=0}^{2} p\big(g_{im}|x_{im} = a, \epsilon\big)p\big(x_{im} = a|z^{\cdot}_{im} = \{k_1, k_2\}, \theta\big). \tag{1}$$

The expression $p\big(g_{im}|x_{im}, \epsilon\big)$ is given by the relevant error model. The second factor in the summand of (1) is given by

$$p\big(x_{im} = a|z^{\cdot}_{im} = \{k_1, k_2\}, \theta\big) = \begin{cases} (1-\theta_{k_1m})(1-\theta_{k_2m}), & a = 0, \\ \theta_{k_1m}(1-\theta_{k_2m}) + \theta_{k_2m}(1-\theta_{k_1m}), & a = 1, \\ \theta_{k_1m}\theta_{k_2m}, & a = 2. \end{cases}$$

Then the probability of the data for one individual may be calculated as

$$p(g_i|\nu,\epsilon) = \sum_{k_1=1}^{K}\sum_{k_2=1}^{K} \phi^i_{\nu,\epsilon}(M,\{k_1,k_2\}),$$

for $i = 1,\ldots,n$.

The backwards recursion is

$$\begin{aligned}\beta^i_{\nu,\epsilon}(m-1,\{k'_1,k'_2\}) &= p(j_{im}=0|\nu)p(g_{im}|z^{\cdot}_{im}=\{k'_1,k'_2\},\nu,\epsilon)\beta^i_{\nu,\epsilon}(m,\{k'_1,k'_2\})\\ &+ \frac{p(j_{im}=1|\nu)}{2}\Bigg(\sum_{k=1}^{K} p(g_{im}|z^{\cdot}_{im}=\{k'_1,k\},\nu,\epsilon)\beta^i_{\nu,\epsilon}(m,\{k'_1,k\})\alpha_{km}\\ &+ \sum_{k=1}^{K} p(g_{im}|z^{\cdot}_{im}=\{k,k'_2\},\nu,\epsilon)\beta^i_{\nu,\epsilon}(m,\{k,k'_2\})\alpha_{km}\Bigg)\\ &+ p(j_{im}=2|\nu)\sum_{k_1=1}^{K}\sum_{k_2=1}^{K} p(g_{im}|z^{\cdot}_{im}=\{k_1,k_2\},\nu,\epsilon)\\ &\times \beta^i_{\nu,\epsilon}(m,\{k_1,k_2\})\alpha_{k_1m}\alpha_{k_2m},\end{aligned}$$

for $m = 2,\ldots,M$, and with $\beta^i_{\nu,\epsilon}(M,\{k_1,k_2\}) := 1$, for all k_1, k_2. For the parameter estimators, we require the following:

$$p(z^{\cdot}_{im}=\{k_1,k_2\}|g_i,\nu,\epsilon) \propto \phi^i_{\nu,\epsilon}(m,\{k_1,k_2\})2^{I_{\{k_1\neq k_2\}}}\beta^i_{\nu,\epsilon}(m,\{k_1,k_2\}), \tag{2}$$

with the constraint that $\sum_{k_1=1}^{K}\sum_{k_2=k_1}^{K} p(z^{\cdot}_{im}=\{k_1,k_2\}|g_i,\nu,\epsilon) = 1$.

2 EM Algorithm

Here we present estimators for α, θ, r, and ϵ, based on the 4-parameter error model (given in Methods), allowing error rates to vary by SNP.

Let $Q\big(\nu,\epsilon|\nu^*,\epsilon^*\big)$ denote $E_{\nu^*,\epsilon^*}\log\Big[p\big(g,x,z,j|\alpha,\theta,r,\epsilon\big)|g\Big]$, the expected complete-data loglikelihood, where the complete-data likelihood is given by

$$p\big(g,x,z,j|\alpha,\theta,r,\epsilon\big)=\prod_{i=1}^{n}p\big(g_i,x_i,z_i,j_i|\alpha,\theta,r,\epsilon\big),$$

with

$$p\big(g_i,x_i,z_i,j_i|\alpha,\theta,r,\epsilon\big)=p\big(g_i|x_i,\epsilon\big)p\big(x_i|z_i,\theta\big)p\big(z_i|j_i\big)p\big(j_i|\alpha,r\big).$$

Setting the partial derivatives of Q to zero and solving for $(\alpha,\theta,r,\epsilon)$ leads to the following estimators:

$$\widehat{\alpha}_{km}=\frac{\sum_i E_{\nu^*,\epsilon^*}\big[j_{imk}|g\big]}{\sum_i\sum_{k'}E_{\nu^*,\epsilon^*}\big[j_{imk'}|g\big]},$$

$$\widehat{r}_m=\frac{\sum_i\sum_k E_{\nu^*,\epsilon^*}\big[j_{imk}|g\big]}{2n},$$

$$\widehat{\theta}_{km}=\frac{A}{B},$$

where

$$A=\sum_i\sum_{k'}p\big(z^{\cdot}_{im}=\{k,k'\}|g_i,\nu^*,\epsilon^*\big)2^{I_{\{k'=k\}}}\left[\left(\frac{\theta^*_{km}\left(1-\theta^*_{k'm}\right)}{\theta^*_{km}\left(1-\theta^*_{k'm}\right)+\theta^*_{k'm}\left(1-\theta^*_{km}\right)}\right)\times\right.$$
$$\left.p\big(x_{im}=1|g,\nu^*,\epsilon^*\big)+p\big(x_{im}=2|g,\nu^*,\epsilon^*\big)\right],\text{ and}$$

$$B=\sum_i\sum_{k'}p\big(z^{\cdot}_{im}=\{k,k'\}|g_i,\nu^*,\epsilon^*\big)2^{I_{\{k'=k\}}}.$$

Given error rates ϵ, we assume the "transitions" from the true genotypes x to observed genotypes g occur marginally at each SNP, according to a multinomial distribution. We apply a prior distribution to ϵ, specifically assuming that ϵ^0 and ϵ^2 are independently distributed as $Beta\,(a,b)$ and $(\epsilon^{10},1-\epsilon^{10}-\epsilon^{12},\epsilon^{12})$ are distributed as $Dirichlet\,(a,b,a)$, where $a=\frac{9}{10}$ and $b=2$. At each iteration of the EM, we obtain the posterior mode of the distribution of ϵ, which leads to the following estimators:

$$\widehat{\epsilon^0}_m=\frac{\sum_i p\big(x_{im}=0|g,\nu^*,\epsilon^*\big)I_{g_{im}=1}+a-1}{\sum_i p\big(x_{im}=0|g,\nu^*,\epsilon^*\big)+a+b-2},$$

$$\widehat{\epsilon^{10}}_m=\frac{\sum_i p\big(x_{im}=1|g,\nu^*,\epsilon^*\big)I_{g_{im}=0}+a-1}{\sum_i p\big(x_{im}=1|g,\nu^*,\epsilon^*\big)+2a+b-3}.$$

Expressions are given for $\widehat{\epsilon^0}_m$ and $\widehat{\epsilon^{10}}_m$. From these, analogous expressions may be obtained for $\widehat{\epsilon^2}_m$ and $\widehat{\epsilon^{12}}_m$ by appropriate substitution.

Estimators for α and r depend on the expected number of jumps in an interval to particular clusters (j_{imk}). These expectations are given by

$$\begin{aligned} E_{\nu,\epsilon}\big[j_{imk}|g\big] = \frac{\alpha_{km}}{p\big(g_i|\nu,\epsilon\big)} \sum_{k'=1}^{K} \Bigg[& p\big(j_{im}=1|r\big) \sum_{k''=1}^{K} \phi^i_{\nu,\epsilon}\big(m-1,\{k',k''\}\big) \\ & + 2p\big(j_{im}=2|r\big)p\big(g_{i(\leq m-1)}|\nu,\epsilon\big)\alpha_{k'm}\Bigg] \\ & \times p\big(g_{im}|z^{\cdot}_{im}=\{k,k'\},\theta,\epsilon\big)\beta^i_{\nu,\epsilon}\big(m,\{k,k'\}\big), \end{aligned}$$

for $m = 1, \ldots, M$ and $k = 1, \ldots, K$.

Finally we give the following formula for computing $p\,(x_{im}|g,\nu)$, which allows computation of the expected number of errors, given the observed data:

$$\begin{aligned} p\big(x_{im}|g_i,\nu,\epsilon\big) \propto p\big(g_{im}|x_{im},\epsilon\big) \sum_{k_1=1}^{K} \sum_{k_2=k_1}^{K} & p\big(x_{im}|z^{\cdot}_{im}=\{k_1,k_2\},\theta\big)\times \\ & p\big(g_{i(<m)}, z^{\cdot}_{im}=\{k_1,k_2\}|\nu,\epsilon\big)\beta^i_{\nu,\epsilon}\big(m,\{k_1,k_2\}\big), \end{aligned}$$

with the constraint $\sum_{a=0}^{2} p\big(x_{im}=a|\cdot\big) = 1$, and where

$$\begin{aligned} p\big(g_{i(<m)}, z^{\cdot}_{im}=\{k_1,k_2\}|\nu,\epsilon\big) = & \, 2^{I_{\{k_1\neq k_2\}}}\phi^i_{\nu,\epsilon}\big(m-1,\{k_1,k_2\}\big)p\big(j_{im}=0|r\big)+ \\ & \Bigg(\alpha_{k_1 m}\sum_{k'=1}^{K} 2^{I_{\{k'\neq k_2\}}}\phi^i_{\nu,\epsilon}\big(m-1,\{k',k_2\}\big)+ \\ & \alpha_{k_2 m}\sum_{k'=1}^{K} 2^{I_{\{k'\neq k_1\}}}\phi^i_{\nu,\epsilon}\big(m-1,\{k',k_1\}\big)\Bigg)\frac{p\big(j_{im}=1|r\big)}{2}+ \\ & 2^{I_{\{k_1\neq k_2\}}}\alpha_{k_1 m}\alpha_{k_2 m}p\big(g_{i(\leq m-1)}|\nu,\epsilon\big)p\big(j_{im}=2|r\big). \end{aligned}$$

This EM algorithm is $\mathcal{O}(K^2Mn)$, linear in the amount of data and K^2.

3 Sensitivity to choice of prior and number of clusters

Here we investigate the effects of various combinations of parameters, in particular the prior distributions for the error rates ϵ and the number of assumed clusters K (Table 1). Discrepancies are tabulated between genotype calls in the HapMap database and the duplicate genotypes calls obtained from the Affymetrix platform (see Methods in main text). Here, as for comparisons presented in the main text, we assume the HapMap genotype calls are correct, which, although is not uniformly the case, gives a first approximation to the accuracy of corrections.

No. clusters (K) /	% Reduction (% Attempted)									
	HapMap MIs				HapMap/Affy. discrepancies					
(a, b)	CEU		YRI		CEU		YRI		JPT+CHB	
K = 5										
$(1, 1)$	1	(.61)	16	(.60)	-41.7	(.301)	-40.6	(.326)	-25.0	(.238)
$(\frac{9}{10}, 2)$	41	(.32)	40	(.37)	-1.3	(.157)	-6.1	(.166)	0.3	(.140)
$(\frac{9}{10}, 20)$	24	(.09)	29	(.14)	0.1	(.008)	0.3	(.010)	1.7	(.023)
K = 8										
$(1, 1)$	-4	(.52)	15	(.54)	-26.3	(.258)	-30.5	(.289)	-16.6	(.215)
$(\frac{9}{10}, 2)$	39	(.28)	38	(.32)	8.3	(.125)	2.7	(.131)	6.9	(.117)
$(\frac{9}{10}, 20)$	20	(.08)	25	(.11)	0.4	(.005)	0.6	(.005)	2.4	(.018)
K = 12										
$(1, 1)$	-9	(.49)	13	(.50)	-18.0	(.228)	-24.4	(.262)	-11.5	(.197)
$(\frac{9}{10}, 2)$	35	(.25)	36	(.28)	13.3	(.102)	7.6	(.104)	10.8	(.100)
$(\frac{9}{10}, 20)$	18	(.07)	22	(.09)	0.4	(.003)	0.6	(.003)	2.4	(.013)

Table 1: *Percent reduction in MIs and discrepancies from correcting genotypes under various parameters.* We correct all genotypes calls if a posterior probability of a genotype different than what is observed exceeds 0.5. Prior distributions for ϵ are $Beta(a, b)$ and $Dirichlet(a, b, a)$ distributions. Reduction in MIs/discrepancies is calculated as 1 minus the total number of MIs/discrpancies divided by the number of MIs/discrepancies in the original data sets, given as a percentage. The percent corrected, given to the right of each reduction value, is the percentage of genotypes in the original data sets that were corrected at the 0.75 probability threshold. A negative value indicates an *increase* rather than a reduction.

4 Sensitivity of alternate error models

Here we briefly investigate the performance of several alternative error models, which are given below. For the reference model (used in the main text) and models A, B, and C, the error rates are allowed to vary across SNPs, although the subscript for the SNP index is suppressed for clarity.

Reference model. This model is the same as that given in the main text. It has 4 error rates for each SNP.

		observed, g		
		0	1	2
	0	$1-\epsilon^0$	ϵ^0	0
true, x	1	ϵ^{10}	$1-(\epsilon^{10}+\epsilon^{12})$	ϵ^{12}
	2	0	ϵ^2	$1-\epsilon^2,$

A. This model has 2 parameters, with separate error rates for homozygous and heterozygous genotypes. In contrast with the reference model, the error rates for the two types of homozygotes are constrained to be equal, as are the error rates if the true genotype is a heterozygote.

		observed, g		
		0	1	2
	0	$1-\epsilon^0$	ϵ^0	0
true, x	1	$\frac{\epsilon^1}{2}$	$1-\epsilon^1$	$\frac{\epsilon^1}{2}$
	2	0	ϵ^0	$1-\epsilon^0,$

B. Same as (A) above, except here a homozygote may be observed if the truth is a homozygote of the other allele.

		observed, g		
		0	1	2
	0	$1-\epsilon^0$	$\frac{\epsilon^0}{2}$	$\frac{\epsilon^0}{2}$
true, x	1	$\frac{\epsilon^1}{2}$	$1-\epsilon^1$	$\frac{\epsilon^1}{2}$
	2	$\frac{\epsilon^0}{2}$	$\frac{\epsilon^0}{2}$	$1-\epsilon^0,$

C. This is a simple error model, with only one parameter (error rate) per marker. The probability of an error does not depend on the underlying (true) genotype.

		observed, g		
		0	1	2
	0	$1-\epsilon$	$\frac{\epsilon}{2}$	$\frac{\epsilon}{2}$
true, x	1	$\frac{\epsilon}{2}$	$1-\epsilon$	$\frac{\epsilon}{2}$
	2	$\frac{\epsilon}{2}$	$\frac{\epsilon}{2}$	$1-\epsilon,$

D. Same as (C) above, with the constraint that the error rates are constant for all SNPs. (In practice, since parameter estimation was conducted independently among chromosomes, the error rates are constant across SNPs on a single chromosome.)

Comparison of results from different error models. As above, discrepancies are tabulated between genotype calls in the HapMap database and Affymetrix duplicate genotypes. Corrected genotypes are scored assuming the HapMap calls are *correct*.

	HapMap MIs (CEU)		HapMap/Affymetrix Discrepancies	
Reference	35.2	(.250)	13.3	(.102)
A	22.2	(.082)	0.01	(.017)
B	25.3	(.094)	-0.14	(.011)
C	3.2	(.010)	≈ 0	(.002)
D	1.6	(.004)	≈ 0	≈ 0

Table 2: *Percent reduction in MIs and discrepancies from correcting genotypes under alternative error models.* Reduction in MIs/discrepancies is calculated as 1 minus the total number of MIs/discrpancies divided by the number of MIs/discrepancies in the original data sets, given as a percentage. The percent corrected, given to the right of each reduction value, is the percentage of genotypes in the original data sets that were corrected at the 0.75 probability threshold. A negative value indicates an *increase* rather than a reduction.

5 HapMap SNPs and Corrected Genotypes

We list approximately 600 SNPs with high LD error rates ($> 10\%$) and zero discerpancies (http://www-personal.umich.edu/~pscheet/Lderror/). Additionally, this resource contains the SNPs with high estimated LD error rates among the JPT+CHB sample, in addition to corrected genotypes.

6 Large Version of Figures

Here we print larger versions of the intensity plots of Figures 1 and 2 from the main text.

Figure 1. *SNP-specific estimates of number of errors based on LD correlate with number of MIs and discrepancies.* Each plot contains a box corresponding to the number of observed MIs or discrepancies (horizontal axis). The position of the bottom and top of a box relates the first and third quartiles of the estimated number of MIs or discrepancies (vertical axis), with the median displayed as a horizontal line in the middle of each box. The red dotted line indicates equality between the number of estimated errors and observed MIs or discrepancies. *First row*: The total number of expected errors at each SNP, based on LD, was calculated for the HapMap data and plotted against the number of MIs. *Second row*: The total number of expected errors at each SNP, based on LD, was calculated for the Affymetrix data, and plotted against the number of discrepancies between the Affymetrix and HapMap genotype calls. In general, the median and the upper quartile for the number of estimated errors increase with the number of discrepancies. This relationship is rather strong for the upper quartile.

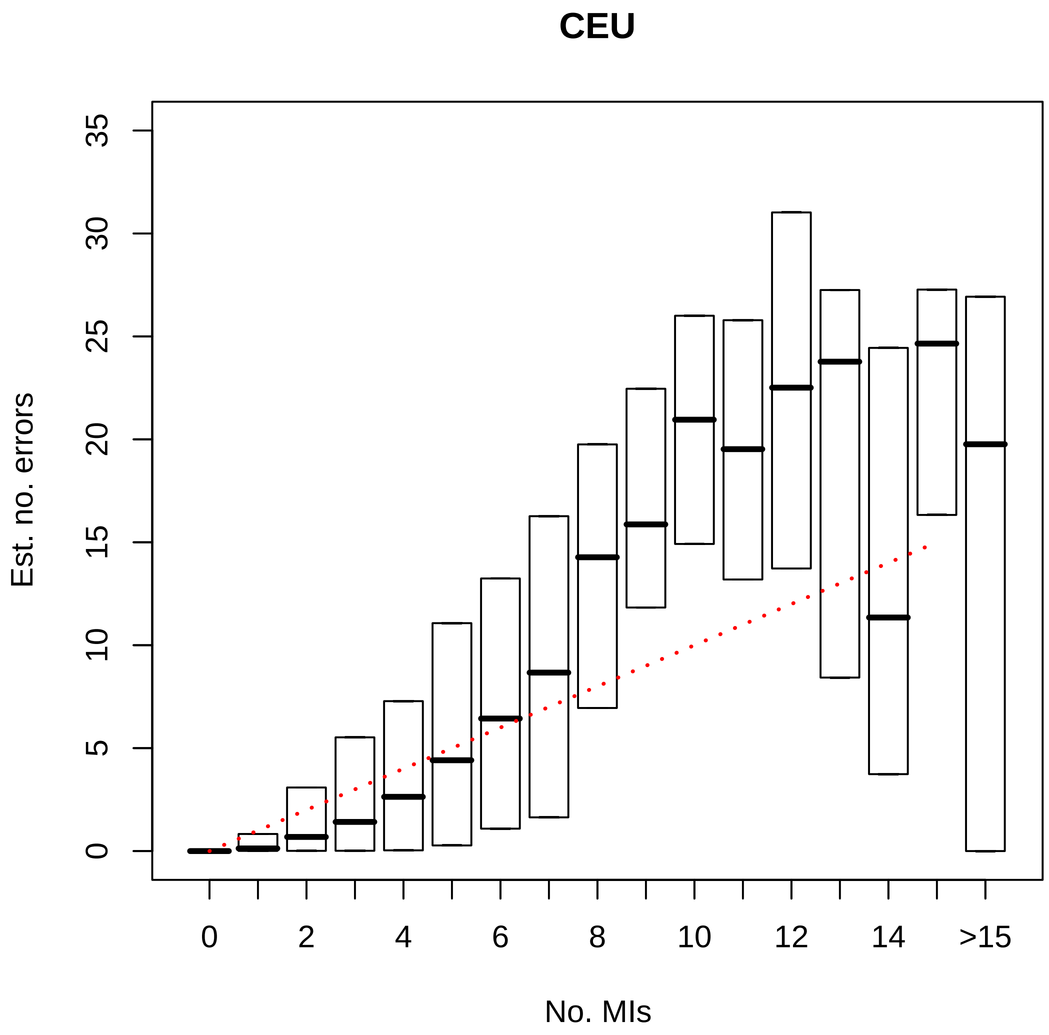

Figure 1 (a). HapMap MIs; CEU

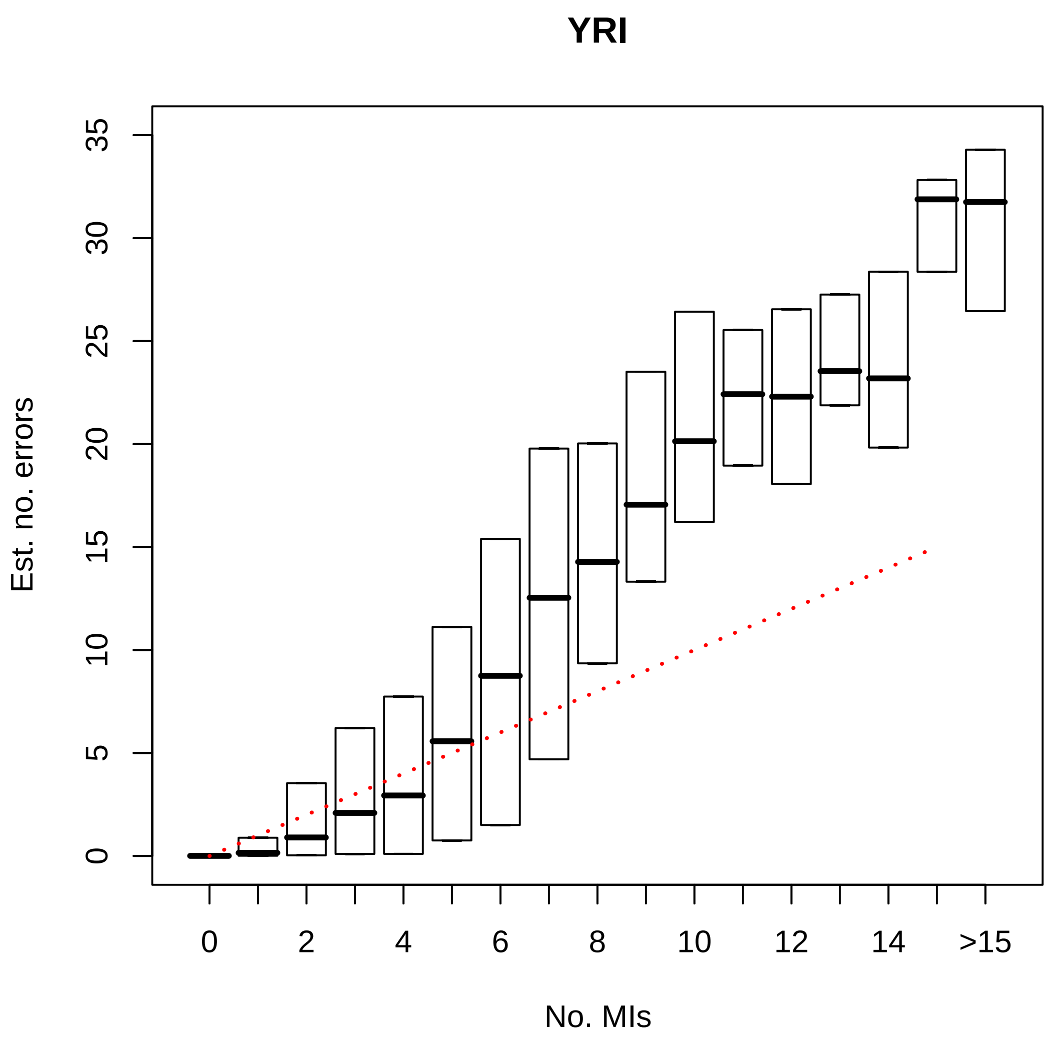

Figure 1 (b). HapMap MIs; YRI

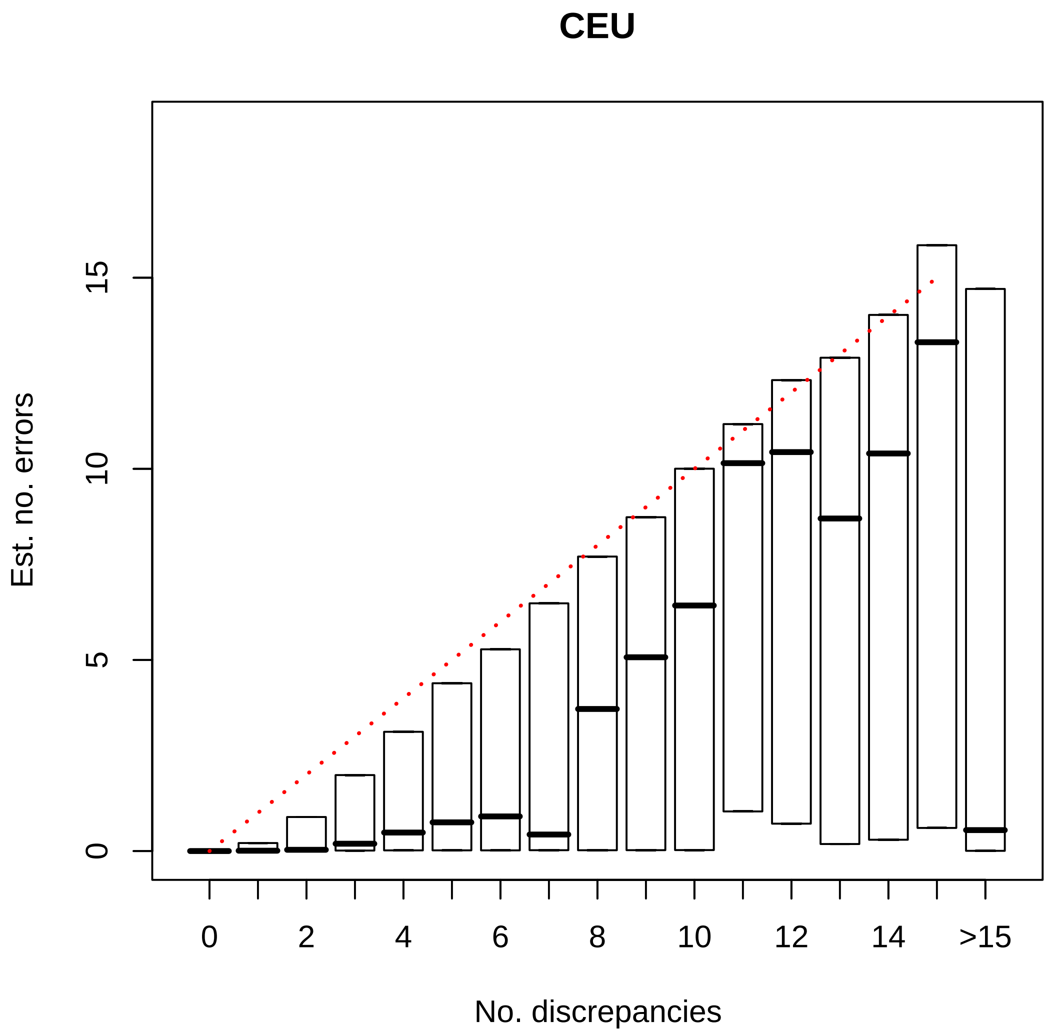

Figure 1 (c). Discrepancies; CEU

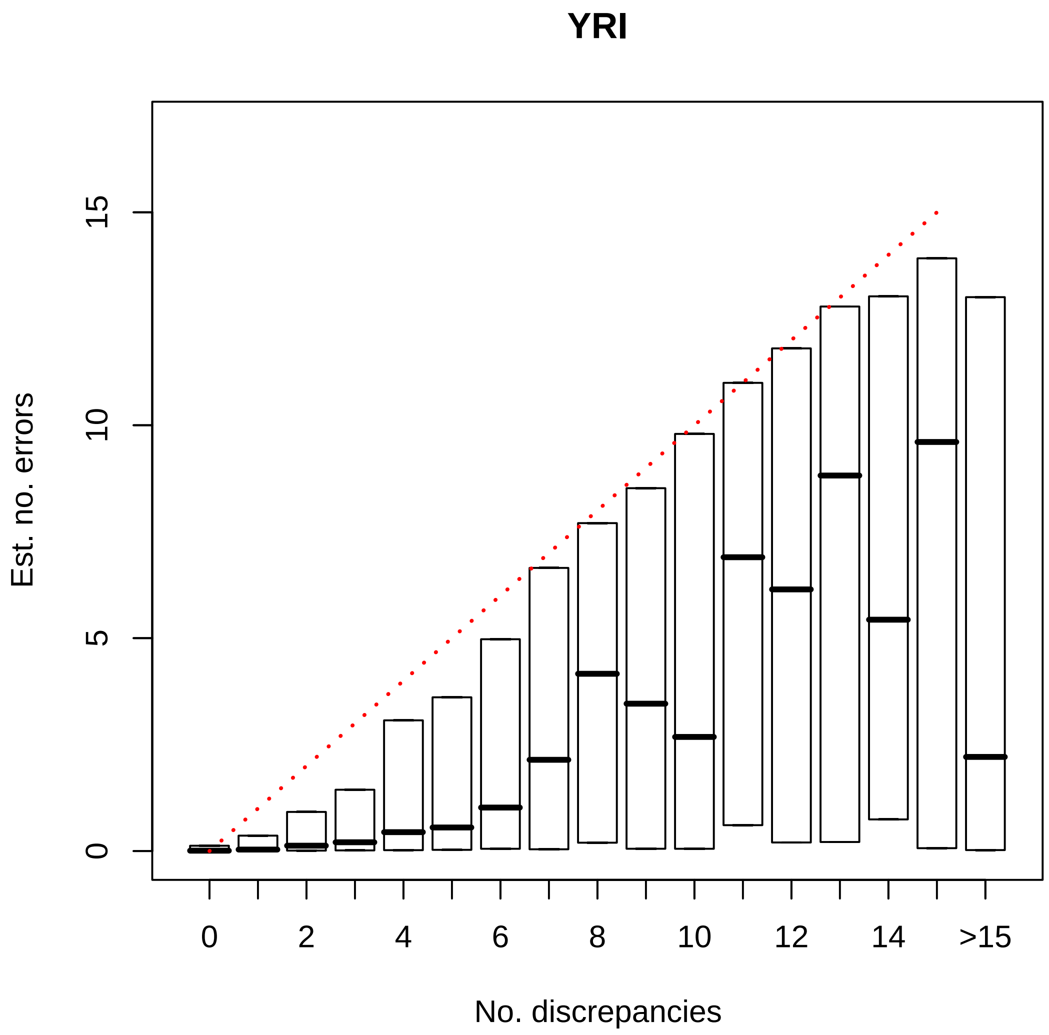

Figure 1 (d). Discrepancies; YRI

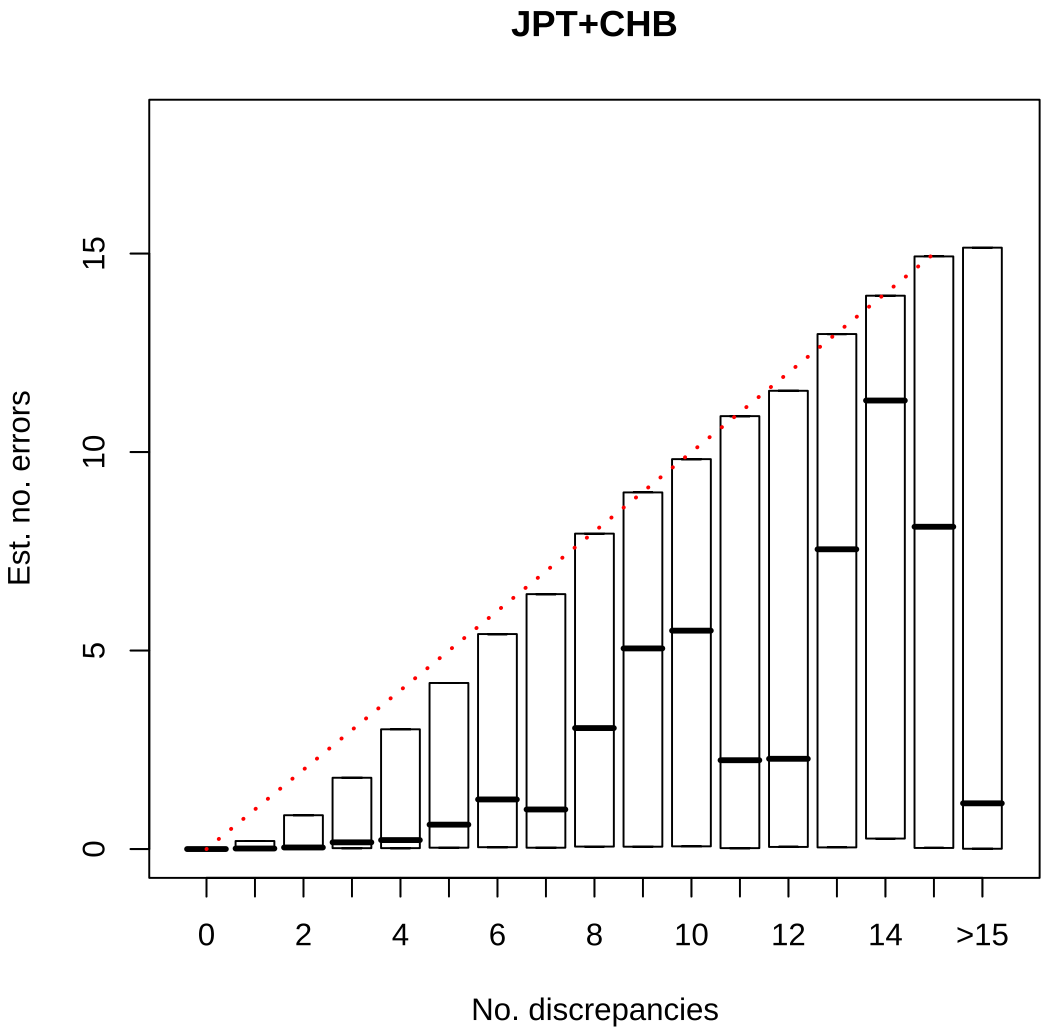

Figure 1 (e). Discrepancies; JPT+CHB

Figure 2. *Example genotype intensity scatter plots from Affymetrix 500K technology on unrelated HapMap samples.* Original calls from the Affymetrix data are indicated by color and shape of the small solid points (homozygotes: ♦, •, heterozygotes: ▲). The larger, open symbols (◊, ○ , △) represent corrected genotype calls from applying our LD-based method to the Affymetrix data. The shapes of the open orange-colored symbols (◊, ○ , △) represent genotype calls according to the HapMap database. LD-based error rate estimates are those obtained from applying the LD-based method to the Affymetrix data. Panel a) shows plots for three SNPs with large numbers of discrepancies between HapMap and Affymetrix calls, but low LD-based error rate estimates and clean intensity plots, with three well-separated clusters. The likely explanation for these results is that the discrepancies are due to errors in the HapMap database, and not the Affymetrix calls on which the LD-based error rates are based. Panel b) shows plots for three SNPs with 0 discrepancies, but high LD-based error rate estimates and unusual intensity plots. The unusual intensity results, combined with the fact that genotypes identified as likely to be incorrect by the LD-based method tend to cluster together, suggests that the high LD-based error rates reflect genuine signal at these SNPs, such as genotyping errors or other anomolies (e.g. Copy Number Variation). This illustrates the potential for the LD-based method to detect problems that duplicate genotyping may miss. Panel c) shows plots for three SNPs with high LD-based error rate estimates, and large numbers of discrepancies, where the intensity plots are relatively clean, but where the genotyping algorithm appears to have done a poor job of clustering the genotypes. In each case the LD-based method succesfully identifies and corrects most of these erroneous genotypes. Although all these examples were chosen to illustrate particular points, they are not atypical in that we saw many other examples of each type of behaviour.

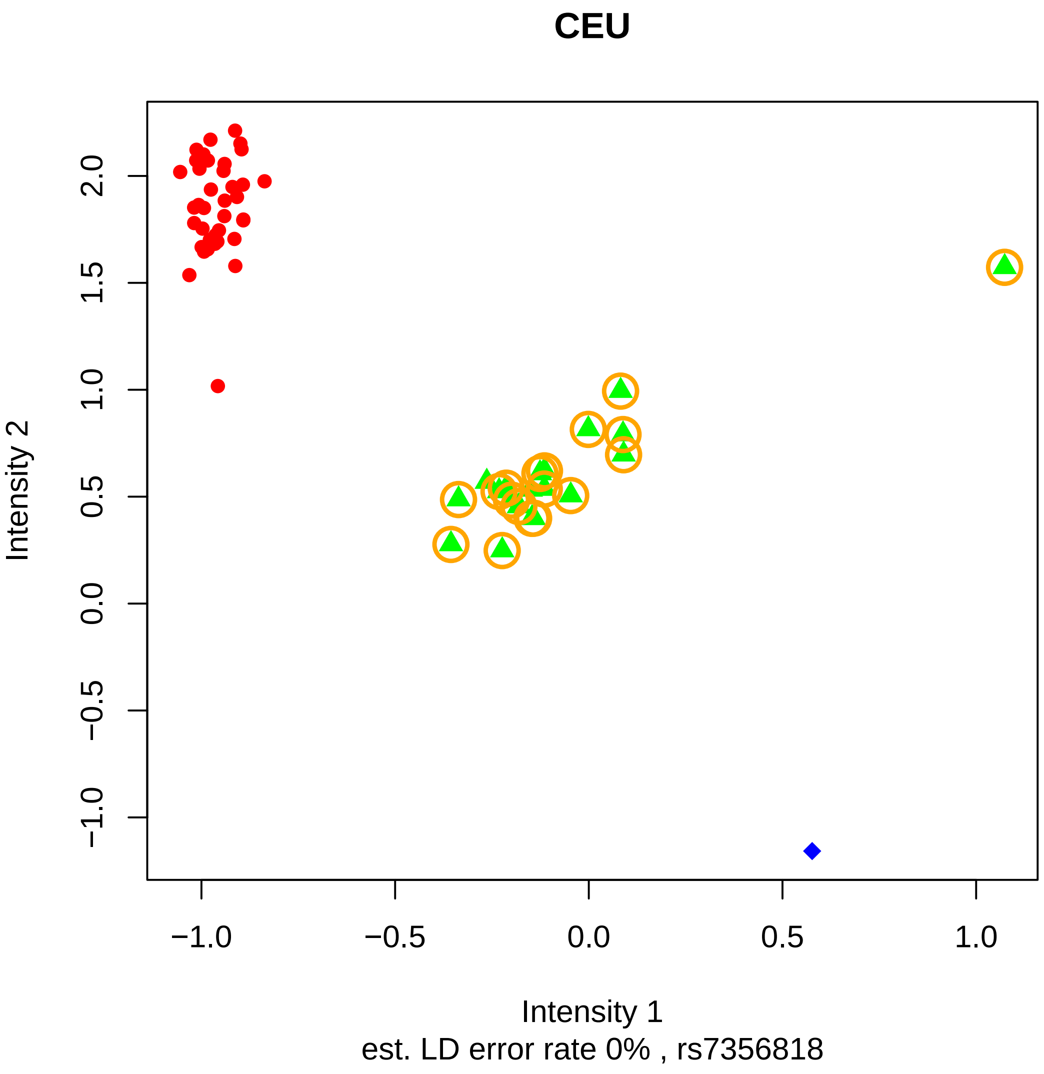

Figure 2 (a) (left)

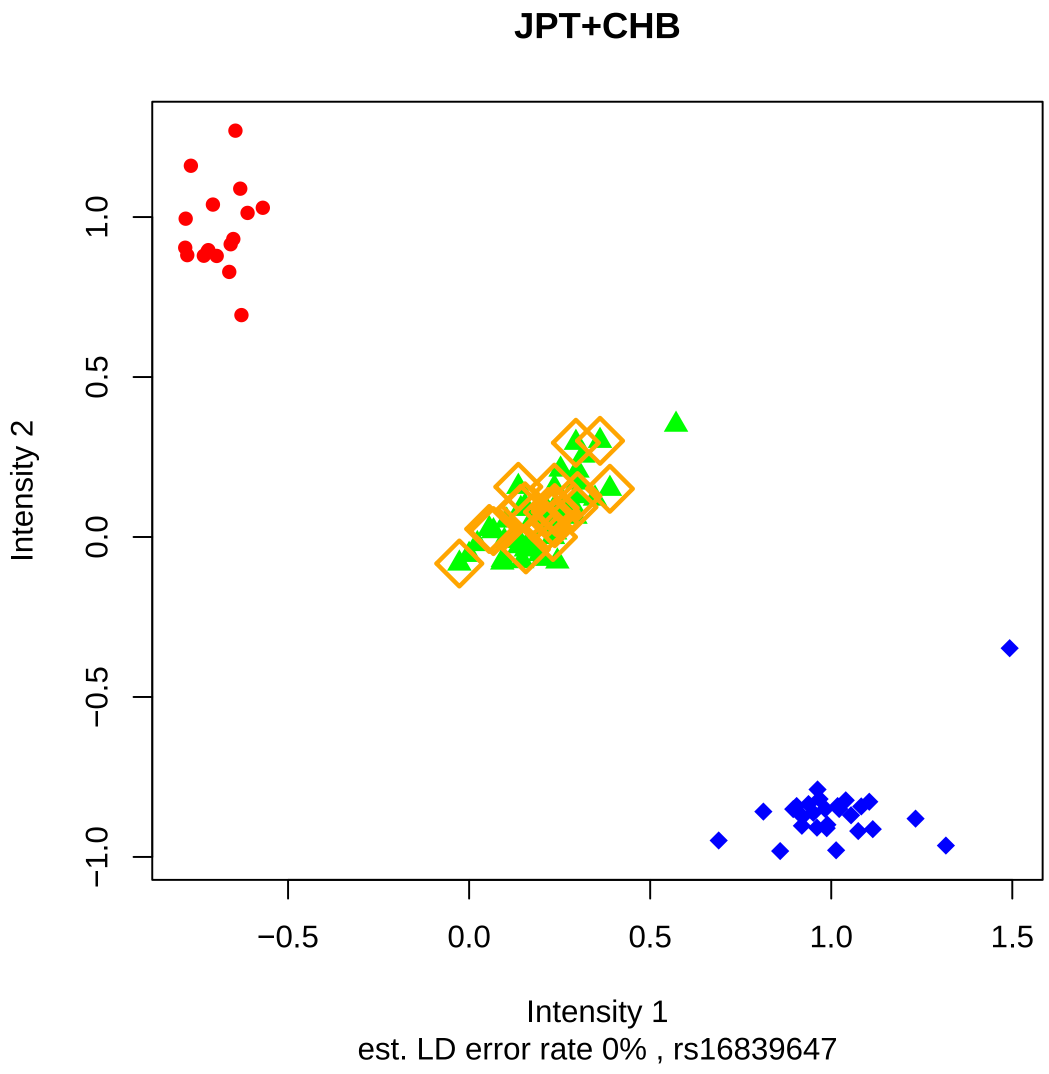

Figure 2 (a) (center)

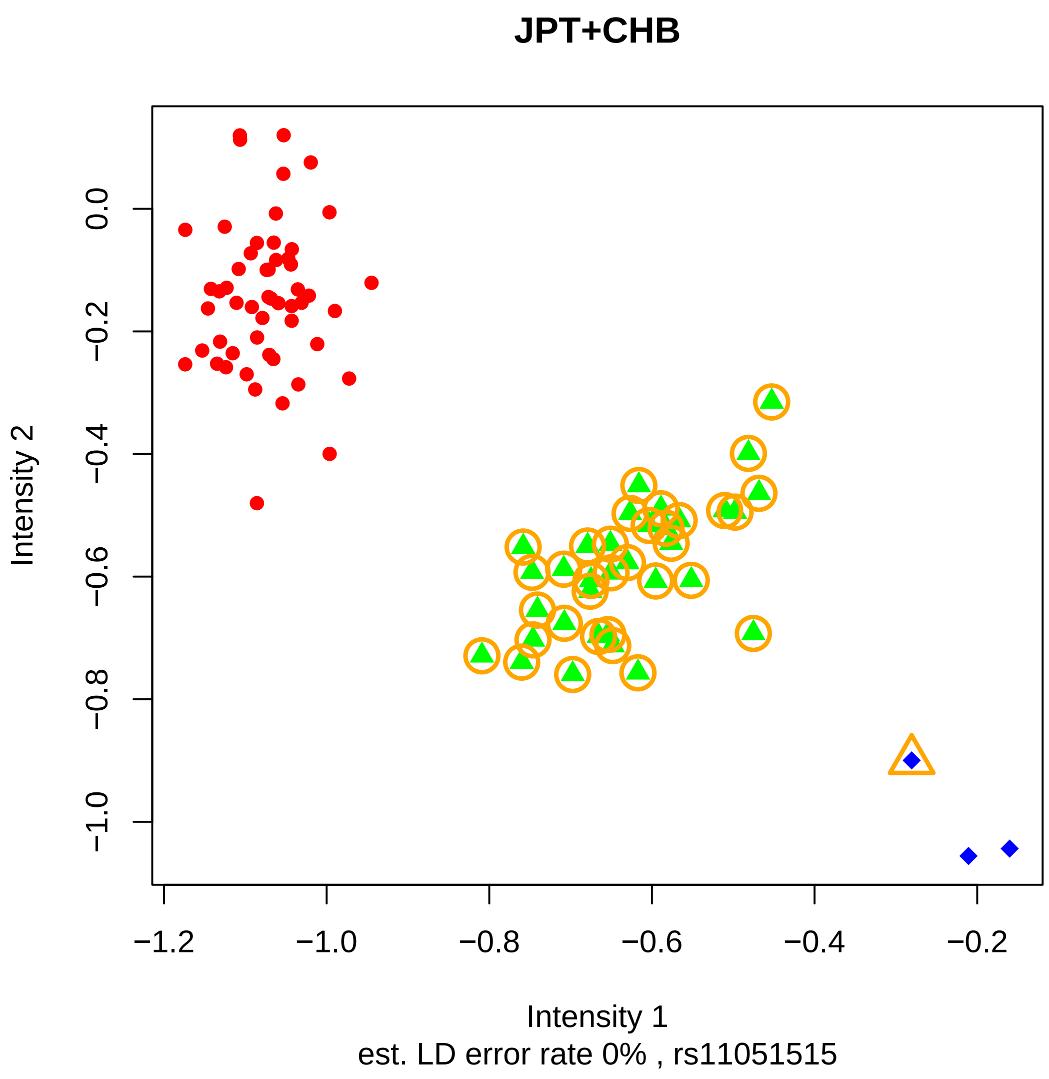

Figure 2 (a) (right)

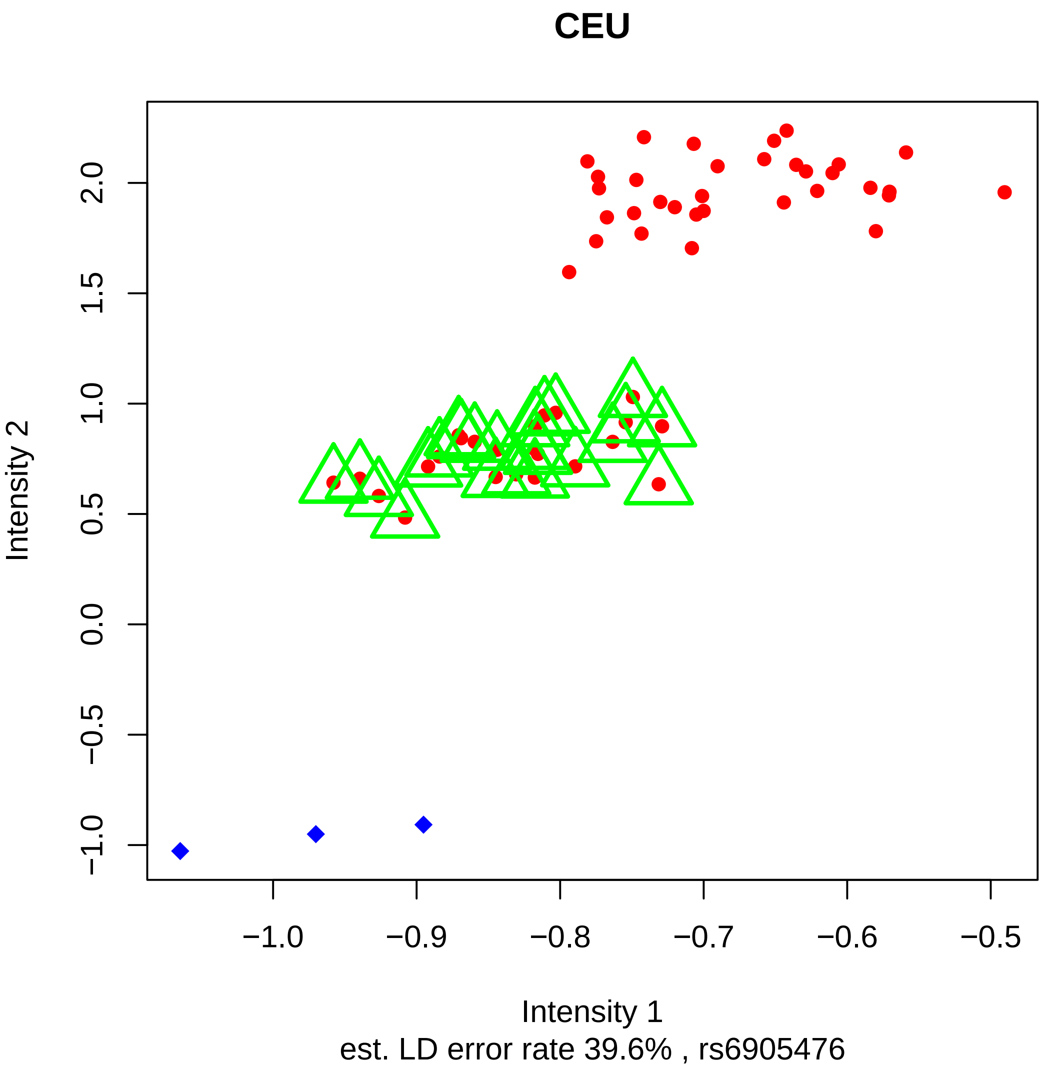

Figure 2 (b) (left)

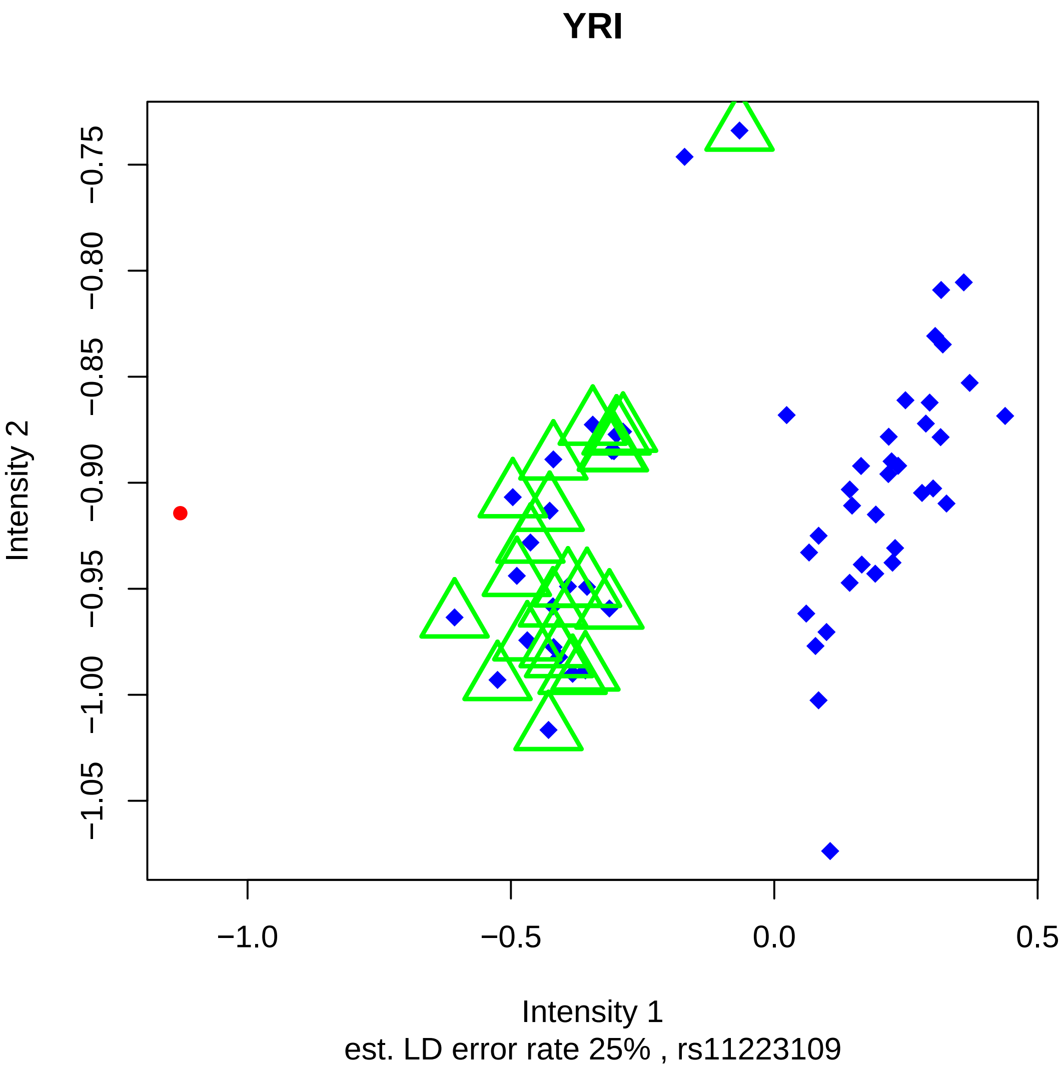

Figure 2 (b) (center)

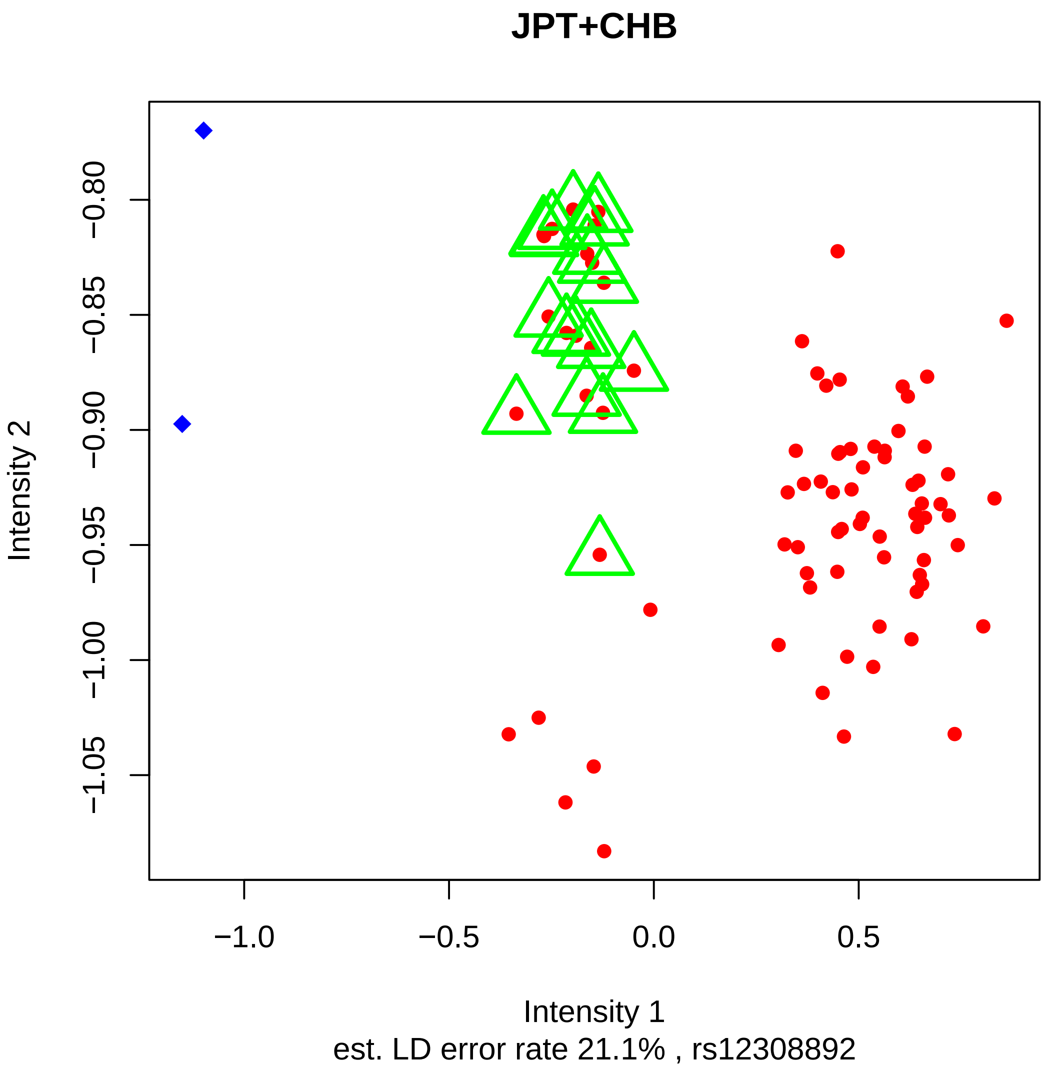

Figure 2 (b) (right)

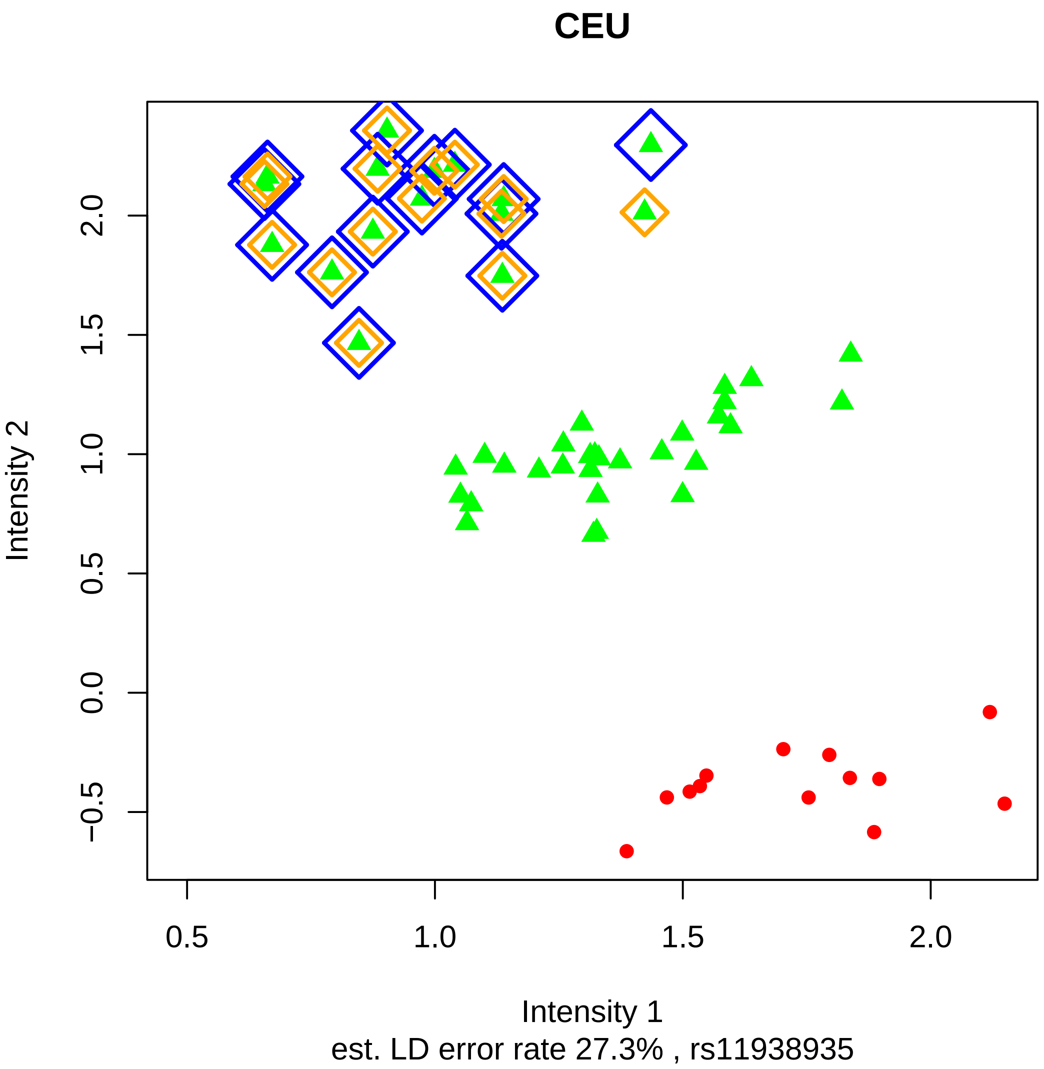

Figure 2 (c) (left)

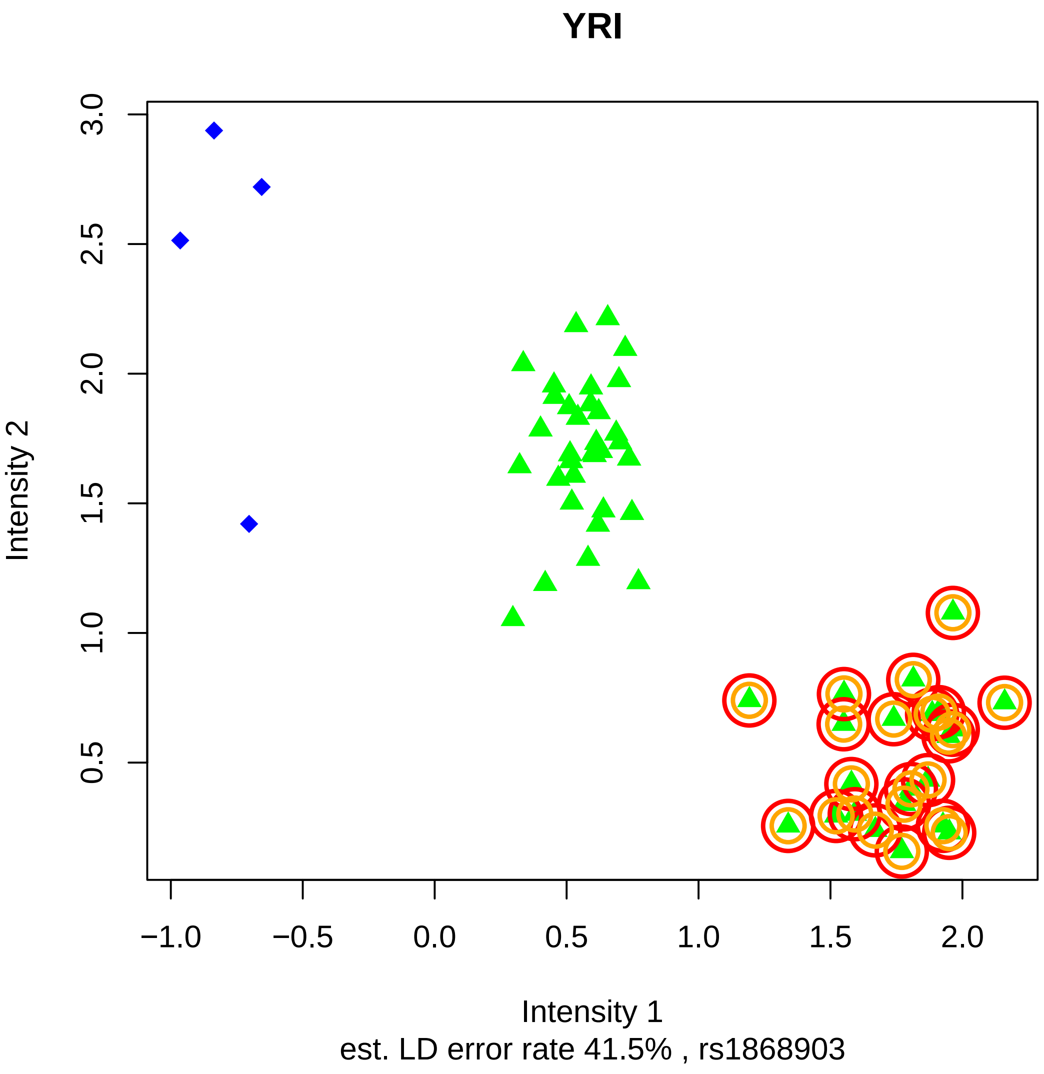

Figure 2 (c) (center)

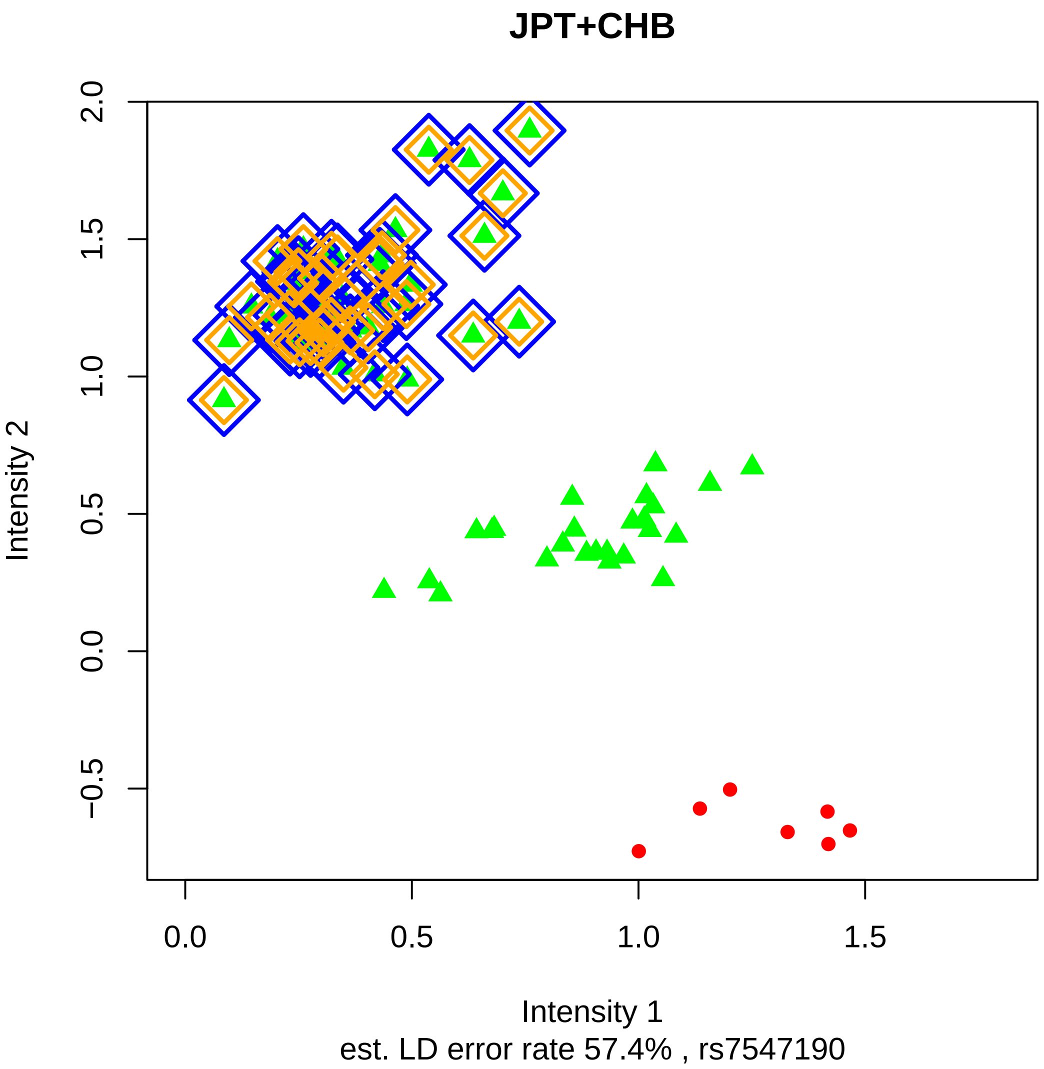

Figure 2 (c) (right)
